# Diving Deeper Into Mechanisms of Acrylamide‐Induced Toxicity: RNA Sequencing Reveals Transcriptomic Alteration and Retrotransposon Expression in *Drosophila melanogaster*

**DOI:** 10.1002/tox.70054

**Published:** 2026-02-23

**Authors:** Oluwabukola Mary Farodoye, Titilayomi Ayomide Otenaike, Daniela Moreira Mombach, Monica Medeiros Silva, Amos Olalekan Abolaji, Elgion Lucio Da Silva Loreto

**Affiliations:** ^1^ Programa de Pós‐Graduação em Genética e Biologia Molecular Universidade Federal do Rio Grande do Sul Porto Alegre Rio Grande do Sul Brazil; ^2^ Departamento de Bioquímica e Biologia Molecular CCNE, Universidade Federal de Santa Maria Santa Maria Rio Grande do Sul Brazil; ^3^ Drosophila Laboratory, Drug Metabolism and Toxicology Unit, Department of Biochemistry, Faculty of Basic Medical Science College of Medicine, University of Ibadan Ibadan Oyo State Nigeria

**Keywords:** acrylamide, *Drosophila melanogaster*, toxicity, transcriptomics, transposable elements

## Abstract

Given the inevitability of human and animal exposure to acrylamide, there is increasing concern regarding its potential health risks. While a number of molecular mechanisms have been proposed, the complexity of acrylamide toxicological pathways and interactions remains incompletely characterized. In this study, we employed a transcriptomic approach to investigate the transcriptional responses of *Drosophila melanogaster* following exposure to acrylamide (100 mg/kg). Our analysis identified 634 differentially expressed genes (DEGs), with 362 upregulated and 272 downregulated. Functional analysis revealed these DEGs are enriched in pathways related to reproduction, detoxification, cellular and metabolic processes, signaling, synaptic formation and organization. Notably, acrylamide exposure upregulated the expression of *tau* and *beta‐amyloid protein precursor‐like* genes, both implicated in Alzheimer's disease pathology. An aversive memory test further demonstrated that acrylamide impaired the short‐term memory of treated flies. Additionally, acrylamide‐induced toxicity altered the expression of nine long terminal repeat retrotransposons, belonging to the *gypsy* and *pao* superfamilies. By exploring the potential role of transposable element activity in acrylamide‐mediated toxicity, this study provides novel insights into the molecular mechanisms underlying its effects. Collectively, these findings offer a more comprehensive understanding of the mechanisms and pathways associated with the toxic action and detoxification of acrylamide in *D. melanogaster.*

## 1 Introduction

Public concerns about the harmful effects of chemical substances in food have increased in recent times, due to changes in dietary habits alongside heightened consumption of processed products. Acrylamide, a process‐related contaminant, is of particular concern because of its widespread exposure. First identified in 2002 as a process‐related contaminant, acrylamide is generated in foods containing carbohydrates and amino groups, through the Maillard reaction, during thermal processing at temperatures above 120°C [1]. Additionally, its formation has been reported through the acrolein pathway and endogenous metabolic processes [2]. Human exposure to acrylamide occurs predominantly through oral intake from the diet, with additional contributions from water, inhalation of tobacco smoke, and dermal contact [3]. Before its recognition as a food contaminant, acrylamide was and remains widely used in industrial manufacturing and scientific research [4]. In adults, average dietary intake is estimated at 0.3 to 0.6 μg/kg of body weight/day, while intakes are typically higher in children due to greater caloric intake relative to body weight. However, total intake of acrylamide can be substantially affected by individual dietary habits. This is because more than one‐third of daily calories comes from food containing detectable acrylamide, and frequent consumption can significantly raise exposure [5].

Clinical and experimental studies have demonstrated that acrylamide adversely affects several systems including the gastrointestinal, neurological, immunological, respiratory, reproductive, endocrine and urogenital systems [1, 4, 6, 7]. Classified by the International Agency for Research on Cancer (IARC) as a Group 2A (probable) human carcinogen [8], acrylamide has also been identified as genotoxic, mutagenic and carcinogenic [9]. Multiple studies have outlined a variety of pathways through which acrylamide may induce toxicity, including oxidative stress, apoptosis, inflammation, autophagy, modulation of the brain‐gut axis, direct inhibition of neurotransmission, cellular changes, inhibition of key cellular enzymes, necrosis and mitochondrial dysfunction [10, 11]. Moreover, phenotypic manifestations occurring from acrylamide exposure, documented from human studies include headaches, dyspnea, sensory impairment, and muscle weakness [4]. However, the full toxicity profile of acrylamide and its mode of action remain to be fully elucidated.

Advances in high‐throughput technologies, such as transcriptomics combined with an expanded bioinformatics toolbox, have been adopted to deepen our understanding of complex biological processes, and for large‐scale data analysis to provide answers to different physiological questions. These advancements have provided invaluable resources for gene expression research, facilitating investigations into the functional properties of genes across multiple biological processes, and the identification of novel mechanisms. As such, we employed RNA‐sequencing to identify the mode of action and to elucidate common and unique molecular pathways modulated by dietary exposure to acrylamide.

Recently, transposable elements (TEs) have been implicated in molecular etiology of several diseases through mechanisms such as insertional mutation, DNA recombination, chromosomal rearrangements, modification of gene expression and epigenetic modifications. Earlier dismissed as “junk DNA,” transposable elements (TEs) are mobile repetitive sequences that account for a large part of eukaryotic genomes and are involved in important gene regulatory functions [12]. Data from ongoing research demonstrate that transposable elements respond to environmental factors, suggesting a potential mechanistic between environmental exposure and diseases. For instance, oxidative stress, radiation, and chemotherapy have been shown to alter TEs expression in somatic cells [13, 14, 15]. This TE transposition in somatic tissues has also been associated with aging, cancer and degenerative diseases [16], while heavy metals such as cadmium and arsenic have been reported to influence TE‐host genome interactions, thereby exacerbating their negative impact [17]. Hence, we aim to investigate TE activity and its potential contribution to acrylamide‐mediated toxicity in *Drosophila melanogaster* . *D. melanogaster* is one of the most extensively studied *Drosophila* species, as it offers a great number of genetic tools. It was among the first eukaryotes to have its genome sequenced and annotated in detail, and it features one of the best comprehensively characterized sets of transposable element sequences [18].

The acrylamide concentration used in this study was selected to produce measurable molecular responses within a controlled, acute timeframe. These findings can therefore provide insights into the molecular mechanisms of acute and sub chronic exposure effects that might also underpin the biological effects of typical environmental exposure levels. Thus, the aim of this study is to: (1) employ RNA‐sequencing as a genomic approach to comprehensively characterize the transcriptional profiles of acrylamide‐induced toxicity in a *Drosophila melanogaster* model, (2) identify both common and unique molecular pathways modulated by acrylamide exposure; and (3) investigate TE activity and its potential contribution to acrylamide‐mediated toxicity. Bioinformatic analysis of the data indicates that acrylamide‐mediated effects on the transcriptome involve genes significantly involved in pathways involved in carbohydrate metabolism, lipid metabolism, reproductive processes, oxidoreductase activity, neuropeptide signaling, and xenobiotic metabolic processes. Based on the sequencing results, we further evaluated the impact of acrylamide exposure on short‐term memory, as well as the glucose and triglyceride levels in both treated and untreated flies.

## 2 Methodology

### 2.1 Drosophila Maintenance and Treatment Exposure

Wild type Oregon strain of *Drosophila melanogaster* used in this study was kept at 25°C and cultured on a standard medium containing cornmeal, wheat, yeast, sugar, and ethylparaben (nipagin). For treatment exposure, the monomeric form of acrylamide (which is the same form found in food and to which humans are exposed dietarily) was dissolved in water and administered to the flies orally by stoichiometric addition to their diet. The choice of acrylamide concentration (100 mg/kg) used was based on previous studies from our research group [19]. Adult flies (0–3 days post eclosion) were exposed to diets with or without acrylamide for 5 days, with three replicates per group (control and acrylamide‐100 mg/kg) and 20 flies per replicate (*n* = 20).

### 2.2 Whole‐Tissue RNA‐Extraction

Total RNA was extracted from the flies using TRIzol (Invitrogen, Carlsbad, CA, USA), according to the manufacturer's protocol. The total RNA was analyzed and quantified by agarose gel electrophoresis, a NanoDrop 2000 spectrophotometer (Thermo Fisher Scientific, Waltham, Massachusetts, USA) and a Qubit3.0 Fluorometer (Thermo Fisher Scientific).

### 2.3 Library Preparation and RNA‐Sequencing

To prepare the library, messenger RNA was isolated with Dynabeads mRNA Purification Kit (Invitrogen) from the RNA samples. The mRNA was fragmented with RNase III, using the Ion Total RNA‐Seq Kit v2 (Thermo Fisher Scientific) according to the manufacturer's instructions. Ion Adapter mix v2 and Ion Xpress RNA‐Seq barcode 01–16 kits were used for adapters. The resulting library pools were quantified with the Qubit dsDNA HS Assay Kit (Invitrogen) and quality checked with Ion Sphere Quality Control Kit (Life Technologies). Templating of the Ion 540 sphere particles was prepared using the One Touch 2 Template v3 kit and then sequencing was performed on an Ion Torrent S5 sequencer (Thermo Fisher Scientific) using the Ion 540 Kit‐OT2 and Ion 540 Chip. Data were collected using Torrent Suite v4.0 software and the resulting reads were exported as raw reads in FASTQ format.

### 2.4 Bioinformatics Analysis of Transcriptome Sequence Data

The raw reads quality was first checked using FastQC after which low‐quality sequencing regions and adapter sequences were trimmed using Trimmomatic V0.39 (https://github.com/usadellab/Trimmomatic/releases; [20]). The trimmed read was uploaded to Galaxy platform (version 24.1.3.dev0) ([21]; https://usegalaxy.org/), and used for the subsequent bioinformatic analysis. Read alignment to the *Drosophila melanogaster* genome was carried out using STAR (Galaxy Version 2.7.11a) [22]. The *D. melanogaster* reference genome v6.58 was obtained from FlyBase (http://ftp.flybase.net/genomes/Drosophila_melanogaster/). FeatureCounts software (Galaxy Version2.0.3) [23] was used to count aligned reads. For differential gene expression analysis, the LIMMA programme (Galaxy Version 3.58.1 + galaxy0) was used, where genes with adjusted *p*‐values (*p*_adj_) of less than 0.05 and log_2_FoldChange (log_2_FC) greater than 1 or less than 1 were considered differentially expressed (DEGs).

### 2.5 Transposable Element (TE) Analysis

The TE table count was generated using TEspeX [24], a tool specifically designed to quantify TEs from RNA‐seq data. TEspeX quantifies TEs expression by counting the number of reads mapped to a reference transcriptome comprising TE consensus sequences, along with annotated coding and non‐coding transcripts. TEs for *D. melanogaster*, v6.58, gotten from Flybase were used as reference. DEseq2, (v1.46, [25]) was used for differential expression analysis and differentially expressed TEs with *p*‐value < 0.05 (padj), were considered significant.

### 2.6 Gene Ontology Analysis

To identify the transcriptomic pathways affected by the exposure, the subset of genes that were significantly affected by the acrylamide treatment were subjected to gene ontology (GO) analysis using ShinyGO (v0.82, https://bioinformatics.sdstate.edu/go/). ShinyGO is a web‐based graphical application for enrichment analysis based on annotation based on Gene Ontology [26] and many other sources [27]. The functional enrichment analysis was performed to identify GOs at cellular components, biological processes, and molecular function categories. GO terms with FDR cutoff < 0.05 were defined as being significantly enriched in DEGs. REACTOME pathway enrichment analysis was performed using the web‐based Database for Annotation, Visualization and Integrated Discovery (DAVID) [28]. STRING (Search Tool for the Retrieval of Interacting Genes/Proteins) tool was used to construct protein–protein interaction networks between the genes enriched in the REACTOME pathways. STRING network analysis was performed with a minimum required interaction score of 0.7 using all the active interaction sources. Disconnected nodes were hidden from the visual network but included in the enrichment analysis (v 12.0, https://string‐db.org/, [29]).

### 2.7 Transcription Qualifications of Memory Genes

The transcriptome data was further analyzed using the TPM (Transcripts per Million) method to obtain gene transcription levels for all memory‐related genes in *D. melanogaster* [30]. We created a candidate gene list from the FlyBase dataset by selecting genes annotated with GO:007611 (“learning or memory”) and then identified those that were differentially expressed in acrylamide‐treated libraries compared to control libraries. Transcriptional fold changes (FC) were established based on the TPM values, with genes considered as differentially expressed if the treated group had a change in TPM values greater than “FC = 1” relative to control. Upregulation is defined as an increase and downregulation as a decrease in expression. Furthermore, DEGs with FC greater than two were examined for protein–protein interaction networks and classified according to their associated type, based on both literature and corresponding GO terms from FlyBase: learning (LRN), short‐term memory (STM; GO:0007614), medium‐term memory (MTM; GO:72375), and long‐term memory (LTM; GO:007616).

### 2.8 Aversive Memory Test by Association

The aversive memory test is an established behavioral assay designed to evaluate learning and memory formation based on the association between a conditioned stimulus (CS) and an unconditioned stimulus (US). In this study, the protocol was adapted from Loreto et al. [31] with minor modifications. Twenty‐five flies per group—including those treated with acrylamide as well as untreated controls—were assessed. First was the “training phase,” where the flies were exposed for a period of 4 min to odor A (3‐octanol diluted in mineral oil at a 1:100 ratio), which served as the CS, while simultaneously receiving electric shocks (approximately 50 V) as the negative unconditioned stimulus. To receive the electric shock, flies were placed into a vial lined with an electrifiable copper grid and exposed to approximately 50 V DC electric shock delivered as 5‐s pulses at 10‐s intervals. Following this phase, a 10‐min rest period was implemented. In the subsequent “testing phase,” the flies were placed in an apparatus where they could choose to move toward one of two compartments: one containing the previously experienced odor A, and the other containing a new odor B (4‐methylcyclohexanol diluted in mineral oil at a 1:50 ratio). The 10‐min interval chosen between training and testing phase characterizes a short‐term memory, which can persist for up to 60 min.

It has already been established that the flies do not have any preference for any of these specific odors [32]. To avoid any potential environmental influences on behavior, the experiment was conducted in the dark under a red light. Behavioral performance was quantified using a performance index that considers the proportion of flies choosing the compartment previously associated with the aversive stimulus (odor A) or the side with the odor B. This index thereby indicates the percentage of flies from the groups able to achieve a memory of association between the electric shock and odor A.

### 2.9 Measurement of Body Glucose and Triglycerides Contents

Triglyceride and glucose levels were determined using the Labtest Assays Kit (Labtest Diagnostica, Brazil). Homogenates of treated and untreated flies were prepared in triglyceride measuring buffer (0.02 M TFK [pH 7.4] + 0.5% Tween 20) or glucose measuring buffer (5 mM Tris buffer (pH 6.6, using 10 μL of buffer per mg of fly mass)). Homogenates were centrifuged at 10 500 × g for 3 min and the resulting supernatants were used to determine triglyceride and glucose levels, following the kit's recommended protocol.

### 2.10 Statistical Analysis

Statistical analyses were performed using the unpaired *t‐*test for parametric data, and the Mann–Whitney test for non‐parametric data. Results were expressed as mean ± SEM and analyzed using GraphPad Prism software version 8.0. The level of significance was set as *p ≤ 0.05. For the differential gene expression analysis, genes with adjusted *p*‐values (*p*_adj_) less than 0.05 and log_2_FoldChange (log2FC) greater than 1 or less than −1 were considered differentially expressed (DEGs).

## 3 Results

### 3.1 RNA‐Seq Analysis

**FIGURE 1:**
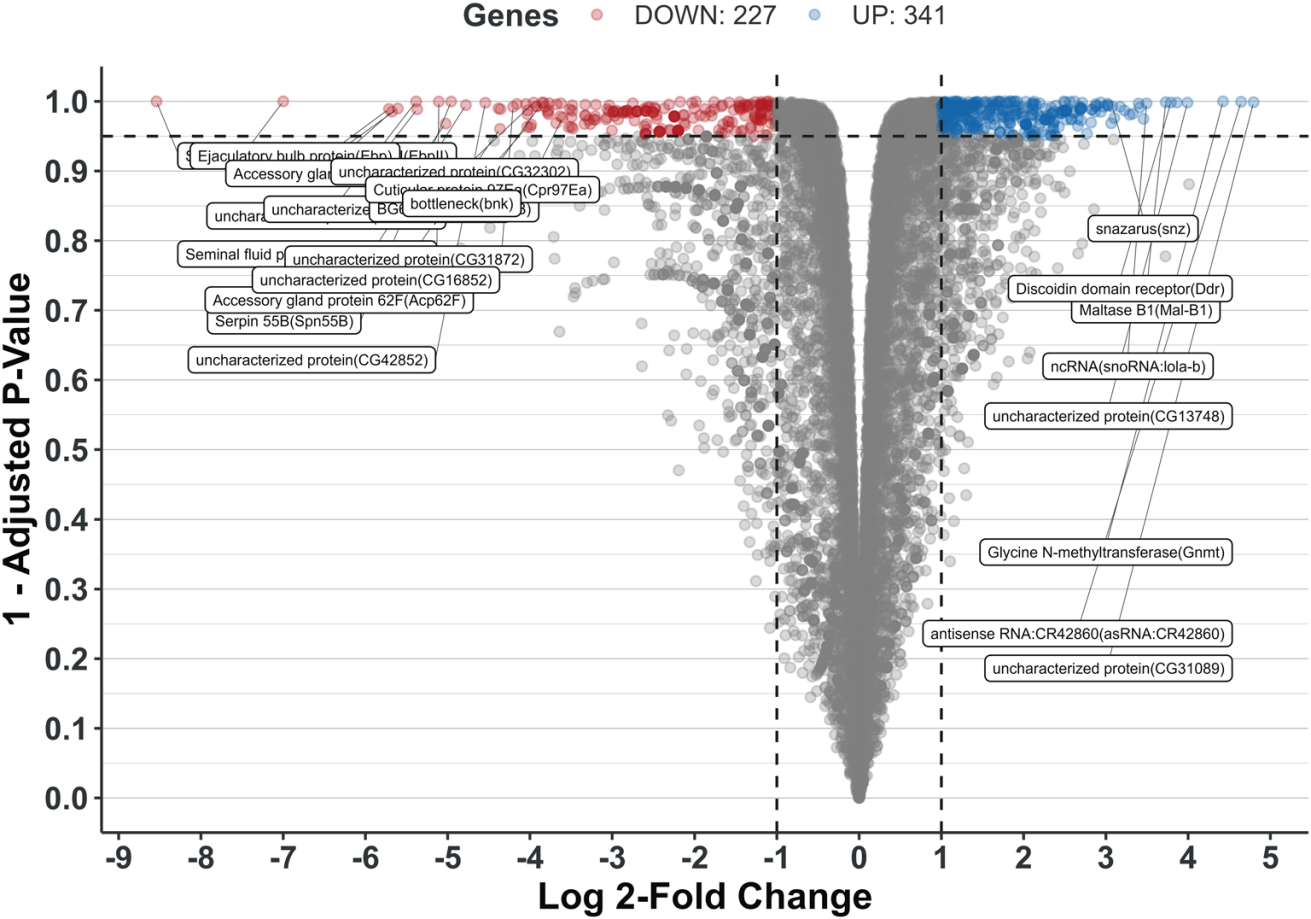
Volcano plot for the gene libraries of the acrylamide‐treated group and the control group of *D. melanogaster* by showing variance in gene expression with respect to log_2_foldchange and *p*‐value (adj). Each dot represents an individual gene. Red dots represent the down‐regulated DEGs, Blue dots represent the upregulated DEGs.

The transcriptional profile of adult fruit flies after 5 days of treatment with or without acrylamide was generated by NGS sequencing using the IonTorrent S5 system. Results from our RNA‐sequencing revealed that exposure to acrylamide induced a significant alteration in the global gene transcription profile of *Drosophila melanogaster*. After the trimming process, 20 167 307 total clean reads were generated for the control group and 19 136 400 for the acrylamide treated group. Over 50% of the total reads were uniquely mapped to the *D. melanogaster* reference genome. Reads were analyzed using LIMMA software on Galaxy to identify differentially expressed genes, and 17 609 genes were found to be transcribed. With *p*_adj_ < 0.05 and log_2_FC greater than 1 or less than 1, 634 genes were found to be differentially expressed, with 362 upregulated and 272 downregulated (Figure 1).

### 3.2 Gene Ontology Enrichment Results of Differentially Expressed Genes

**FIGURE 2:**
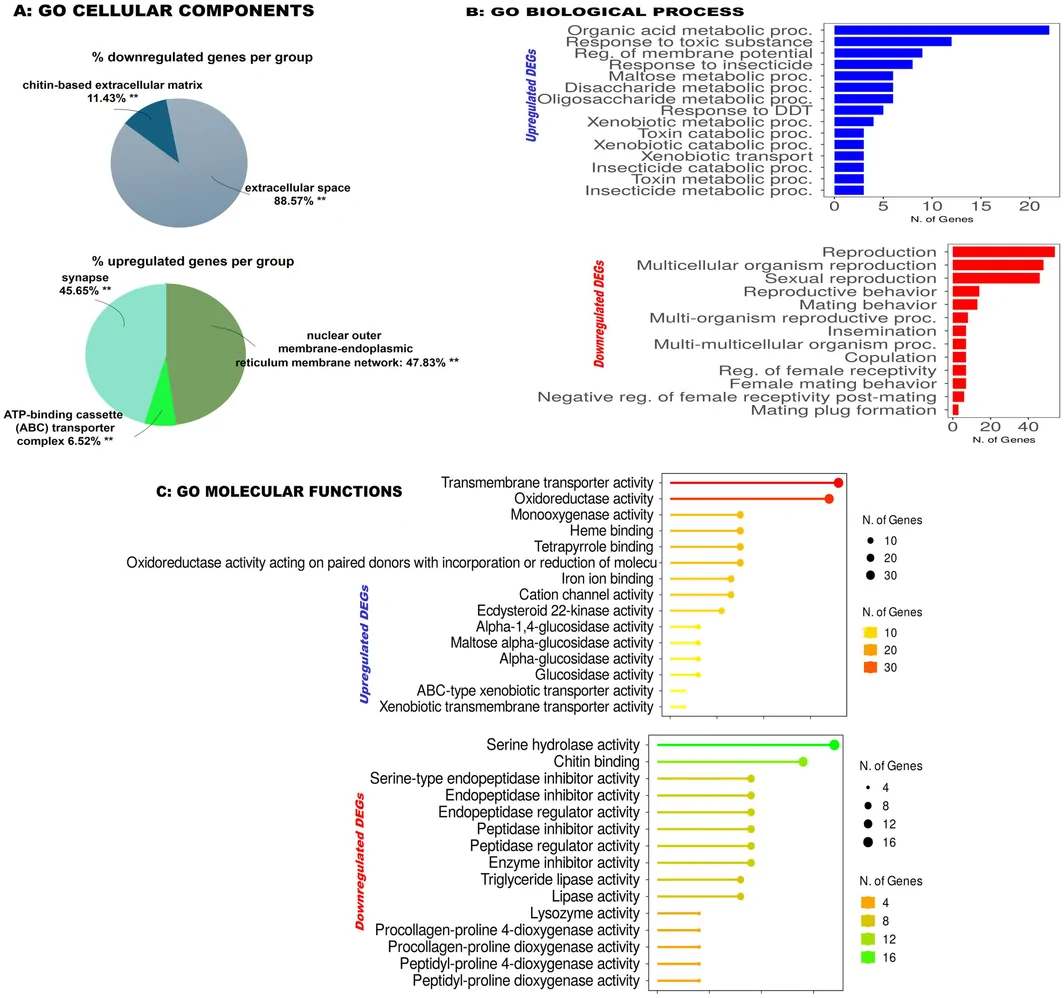
Overview of Gene Ontology (GO) of DEGs in control and acrylamide‐treated datasets. Distribution of GO categories was assigned into three categories: (a) cellular component, (b) biological process, and (c) molecular functions. GO term enrichment was performed using ShinyGO.

Gene ontology enrichment analysis provides information on the functional relation among a large set of genes. Specifically, we used ShinyGO (v0.82), a graphical web gene‐set enrichment tool that creates a functionally organized GO term network as defined by Gene Ontology. GO terms with FDR enrichment < 0.005 were defined as being significantly enriched in DEGs. In GO analysis, the DEGs were divided into three categories: cellular component (CC) (Figure 2a), biological process (BP) (Figure 2b), and molecular function (MF) (Figure 2c). The results showed that the DEGs were significantly enriched in 32 items in BP, 2 items in CC and 30 items in MF for downregulated genes. While 54 items in BP, 69 items in MF and 3 items in CC were significantly enriched for upregulated genes. The top 15 terms in each category are shown in Figure 2 (Full list of the enriched ontology for molecular and biological processes are included in Tables S1 and S2, respectively). “Mating plug formation,” “negative regulation of female receptivity post‐mating,” “insemination,” “lysozyme activity” were some of the subcategories mostly enriched among the 272 downregulated DEGs (Figure 2b). For the 362 upregulated DEGs, “maltose metabolic process,” “toxin catabolic process,” “regulation of membrane potential,” and “ABC‐type‐xenobiotic transporter activity” are some of the enriched terms for GO: BP and GO: MF ontologies (Figure 2b,C). To reveal molecular interaction information and potential metabolism pathways, all DEGs were further mapped to the Reactome database.

### 3.3 Enrichment Results of Reactome Pathways for Differentially Expressed Genes

**FIGURE 3:**
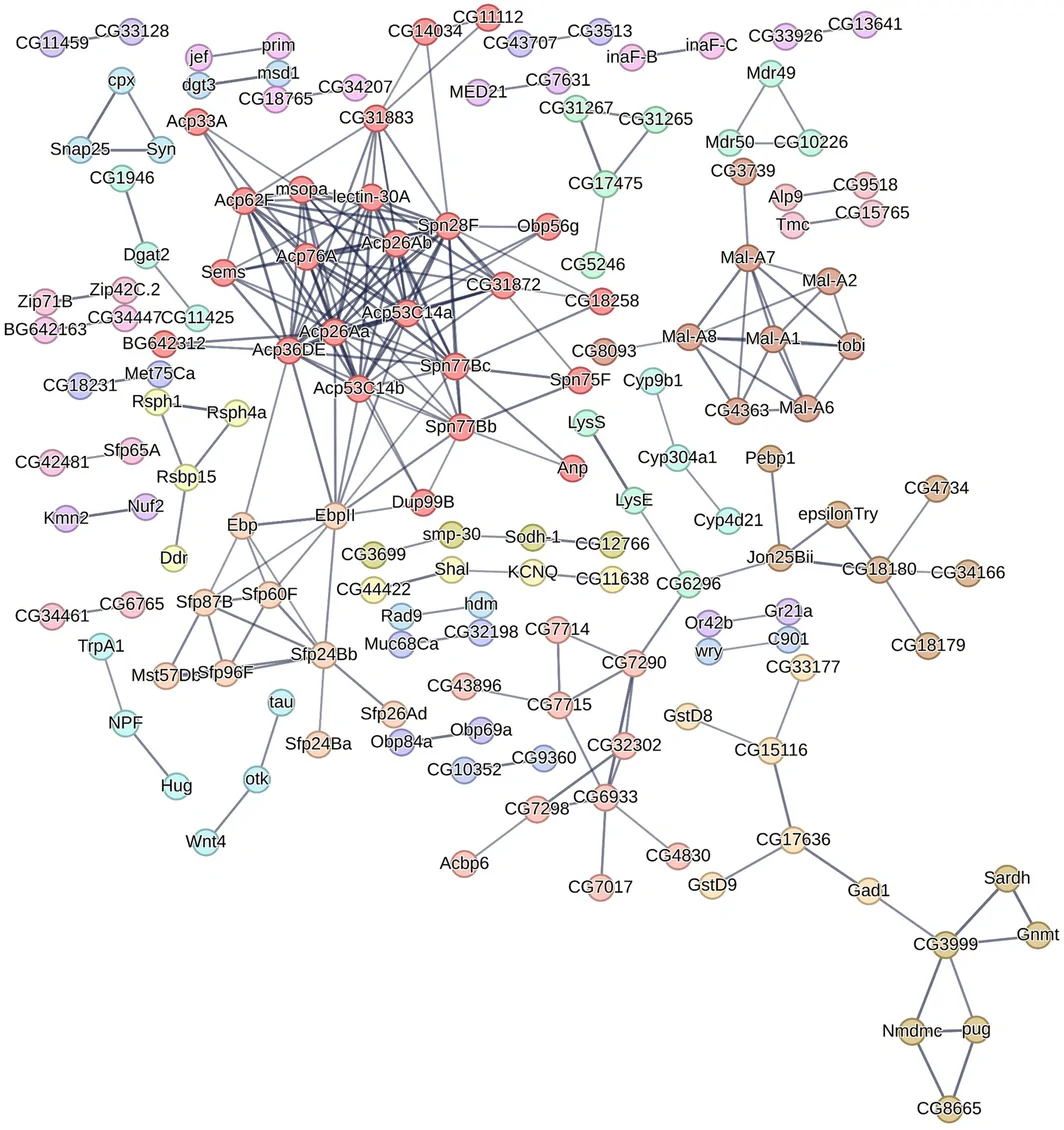
Protein–Protein interaction (PPI) network of differentially expressed genes associated with acrylamide exposure. The PPI network was constructed using STRING with a high‐confidence score threshold of 0.700. Each node represents a protein, and the lines depict interactions between them. Line thickness represents the confidence of interactions between proteins. Functionally related proteins are clustered (indicated by distinct colors) using the Markov Cluster Algorithm (MCL).

Enrichment pathway analysis produced 56 significant Reactome pathways for the DEGs. Table 1 shows the top 20 enriched pathways, based on *p*‐value (< 0.05). Enrichment of 66 genes associated with metabolism indicates that acrylamide has a profound effect on cellular and metabolic processes. Pathways involving drug ADME, paracetamol ADME, Cytochrome P450, and digestion imply that acrylamide is recognized as an exogenous substance, subsequently triggering a series of defense responses in the fly. A high percentage of the total differentially expressed genes were associated with neurotransmission, neuronal system, signaling, and transmission across chemical synapses pathways. Genes related to the insect hormone pathway, folate biosynthesis, and amino acid metabolic processes were also enriched. Table S3 contains the complete list of the significant REACTOME pathways and their associated genes. To gain insight into the functional relationship among differentially expressed genes identified in our study, we constructed a protein–protein (PPI) network using the STRING database (Figure 3). For accurate interaction predictions, the PPI network was generated with a high‐confidence score of 0.7. We also employed the Markov Cluster Algorithm (MCL) to describe groups of functionally related proteins (indicated by specific colors). For instance, this PPI network suggests a coordinated response to acrylamide exposure and that acrylamide disrupts multiple biological processes simultaneously.

**TABLE 1:** Top 20 differentially regulated REACTOME pathways in acrylamide‐treated *D. melanogaster* compared with the control.

S/N	REACTOME pathway term/ID	No of genes	*p*	Fold enrichment
1	R‐DME‐556833~Metabolism of lipids	38	6.25E^−08^	2.558009
2	R‐DME‐1430728~Metabolism	66	8.09E^−07^	1.715594
3	R‐DME‐9748784~Drug ADME	17	4.32E^−06^	3.936317
4	R‐DME‐500792~GPCR ligand binding	12	8.65E^−06^	5.441379
5	R‐DME‐373076~Class A/1 (Rhodopsin‐like receptors)	11	1.59E^−05^	5.700493
6	R‐DME‐196071~Metabolism of steroid hormones	11	2.12E^−05^	5.525093
7	R‐DME‐425407~SLC‐mediated transmembrane transport	19	1.19E^−04^	2.781692
8	R‐DME‐382551~Transport of small molecules	32	1.31E^−04^	2.020783
9	R‐DME‐8978868~Fatty acid metabolism	14	4.06E^−04^	3.152247
10	R‐DME‐8957322~Metabolism of steroids	12	4.53E^−04^	3.56163
11	R‐DME‐112310~Neurotransmitter release cycle	9	4.54E^−04^	4.816959
12	R‐DME‐352230~Amino acid transport across the plasma membrane	8	6.13E^−04^	5.330331
13	R‐DME‐112316~Neuronal System	16	0.001867	2.452453
14	R‐DME‐2142753~Arachidonate metabolism	6	0.001873	6.529655
15	R‐DME‐372790~Signaling by GPCR	14	0.003016	2.53931
16	R‐DME‐1483206~Glycerophospholipid biosynthesis	11	0.0031	3.017908
17	R‐DME‐381426~Regulation of Insulin‐like Growth Factor (IGF) transport and uptake by Insulin‐like Growth Factor Binding Proteins (IGFBPs)	8	0.003614	3.957367
18	R‐DME‐8957275~post‐translational protein phosphorylation	8	0.003614	3.957367
19	R‐DME‐9757110~Prednisone ADME	8	0.003935	3.898302
20	R‐DME‐193144~Estrogen biosynthesis	6	0.004281	5.441379

*Note:* Pathway enrichment analysis was based on Bonferroni‐corrected *p* < 0.005.

### 3.4 Glucose and Triglycerides Level

**FIGURE 4:**
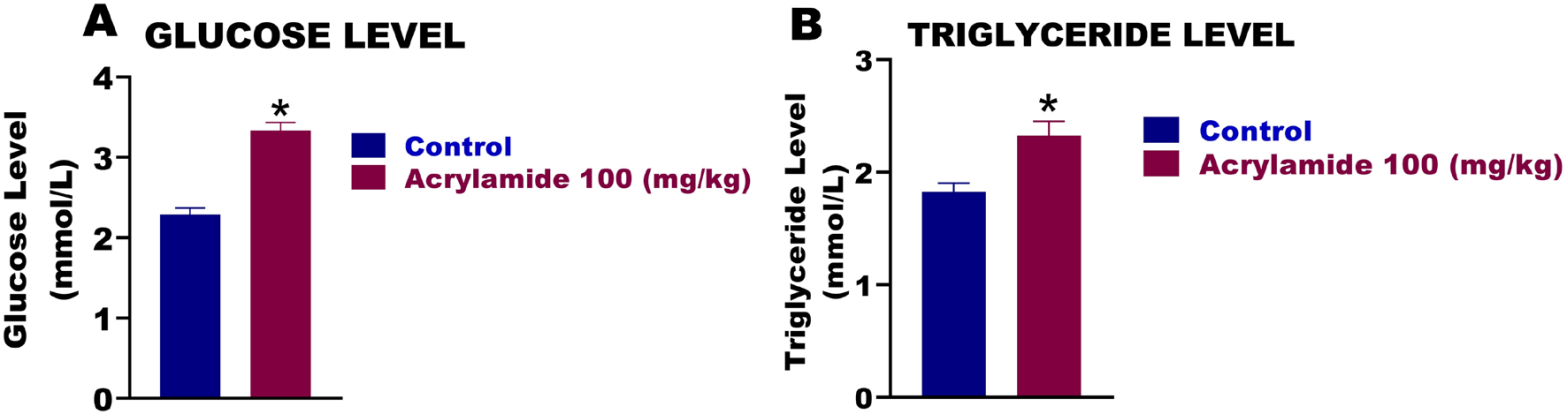
Effect of acrylamide (100 mg/kg) on the glucose and triglyceride level of *D. melanogaster* after 5 days of exposure. Error bars represent the standard error of the mean. Significant differences from the control are indicated by *p < 0.05.

Based on the observed significant expression of genes involved in glucosidase activity, we further assessed the levels of glucose and triglycerides in the flies. A significant increase (*p* < 0.05) was observed in both glucose (Figure 4a) and triglycerides levels (Figure 4b) of acrylamide‐treated flies. Acrylamide‐treated flies had an increase of 1.46‐fold and 1.27‐fold in their glucose and triglyceride content, respectively, compared to the control group (Figure 4).

### 3.5 Memory Test

**FIGURE 5:**
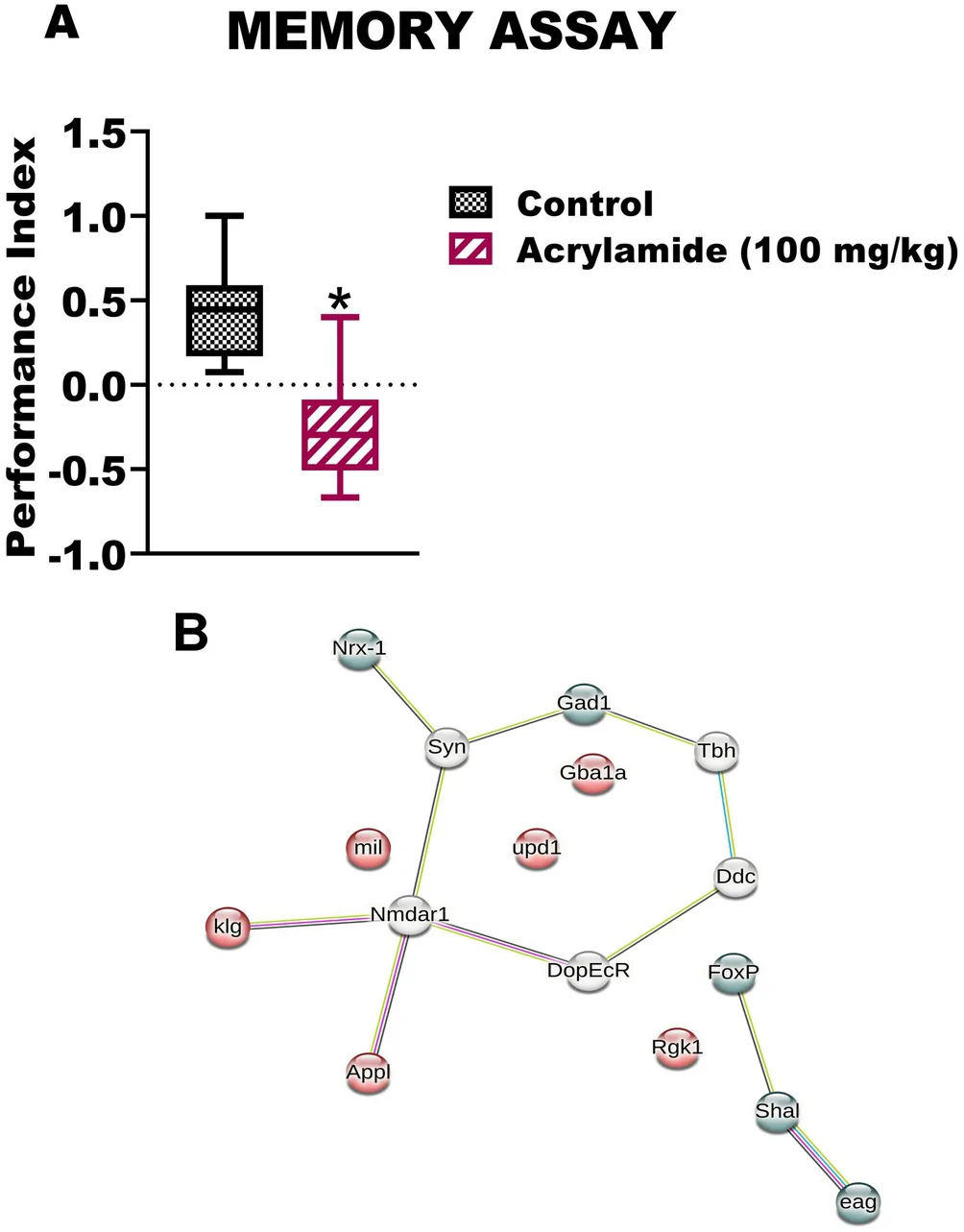
(a) Effects of acrylamide exposure on flies' aversive short‐term memory by association. The test was assessed in flies after 5 days of treatment with acrylamide. Significant differences from the control are indicated by *p < 0.05. (b) Protein–protein interactions of differentially expressed genes involved in learning/memory obtained from acrylamide‐treated and control libraries (FC > 2). Red colors indicate the six genes related to memory (including short‐term, middle‐term, and long‐term), teal color for five genes related to learning (including associative learning), and silver for five genes associated with learning and memory. Each node represents all the proteins produced by a single, protein‐coding gene locus, with line colors indicating interactions based on all active interaction sources on STRING.

Memory loss is a key symptom of some neurodegenerative diseases, including Alzheimer's and other forms of dementia. Given the differential expression of genes associated with AD, we decided to assess the effect of acrylamide intake on memory performance using an aversive memory test. Results from the study showed that acrylamide‐treated flies exhibited significant impairment in short‐term memory, as indicated by the negative performance index of the treated group compared to the control group (−0.26 vs. 0.43, *p* < 0.05; Figure 5a).

### 3.6 Transcription Quantification of Memory‐Related Genes

Further investigation into the impact of acrylamide on cognitive function shows that, of the 177 genes associated with “learning & memory” on FlyBase (GO:007611), 69 genes had a transcriptional fold change > 1 in our dataset and 21 with FC > 2 (Table S4). Subsequent analysis of the genes with FC > 2 showed six genes primarily related to memory (*mil*, *Gba1a*, *Appl*, *klg*, *Rgk1*, *upd1*), five genes related to learning (*Nrx‐1*, *shal*, *Gad1*, *eag*, *FoxP*), and five genes related to learning and memory (*Tbh*, *Syn*, *Nmdar1*, *DopEcr*, *Ddc*). As presented in Table 2, acrylamide caused downregulation of *Gba1a* (FC = −2.30) and *mil* (FC = −2.59), two genes associated with memory while causing upregulation of the other genes. Protein–protein interaction (PPI) network analysis revealed the networks of these key genes associated with the effect of acrylamide on memory (Figure 5b). *N*‐methyl‐D‐aspartate receptor (*NMDAR 1*, 2.32‐fold increase in expression) was identified as a key hub gene from the PPI analysis. *NMDAR 1* interacts with other genes involved in signaling pathways associated with learning and memory, including *Appl*, *klg*, *Syn*, and *DopEcR*, which in turn interact with other genes causing a downstream effect.

**TABLE 2:** List of differentially expressed genes involved in learning/memory obtained from acrylamide‐treated and control libraries (FC > 2).

Gene name	Control TPM mean	Acrylamide TPM mean	Fold change	
*Milkah (mil)*	959.73	372.61	−2.58	*MEMORY*
*Glucocerebrosidase 1a (Gba1a)*	25 577.37	11 329.78	−2.26	*MEMORY*
*beta amyloid protein precursor‐like (Appl)*	3800.52	8728.00	2.30	*MEMORY*
*Klingon (klg)*	1248.15	3314.68	2.66	*MEMORY*
*Rad, Gem/Kir family member 1 (Rgk1)*	573.48	1747.38	3.05	*MEMORY*
*unpaired 1 (upd1)*	282.11	1049.17	3.72	*MEMORY*
*Neurexin 1 (Nrx‐1)*	1822.45	3925.06	2.15	*LEARNING*
*Shaker cognate l (Shal)*	1782.52	3888.27	2.18	*LEARNING*
*Glutamic acid decarboxylase 1 (Gad1)*	7081.17	18 244.43	2.58	*LEARNING*
*ether a go‐go (eag)*	232.03	664.88	2.87	*LEARNING*
*Forkhead box P (FoxP)*	57.36	290.26	5.06	*LEARNING*
*Tyramine beta hydroxylase (Tbh)*	774.86	1688.47	2.18	*MEMORY/LEARNING*
*Synapsin (Syn)*	1361.92	3177.15	2.33	*MEMORY/LEARNING*
*NMDA receptor 1 (Nmdar1)*	444.55	1042.37	2.34	*MEMORY/LEARNING*
*Dopamine/Ecdysteroid receptor (DopEcR)*	1929.87	5061.76	2.62	*MEMORY/LEARNING*
*Dopa decarboxylase (Ddc)*	6796.64	18 357.49	2.70	*MEMORY/LEARNING*

*Note:* Genes are grouped and colored according to their association with either memory alone, learning alone, or both learning and memory. Negative FC indicates downexpression, while positive indicates upregulation.

### 3.7 Differential Expression Analysis of Transposable Elements

**FIGURE 6:**
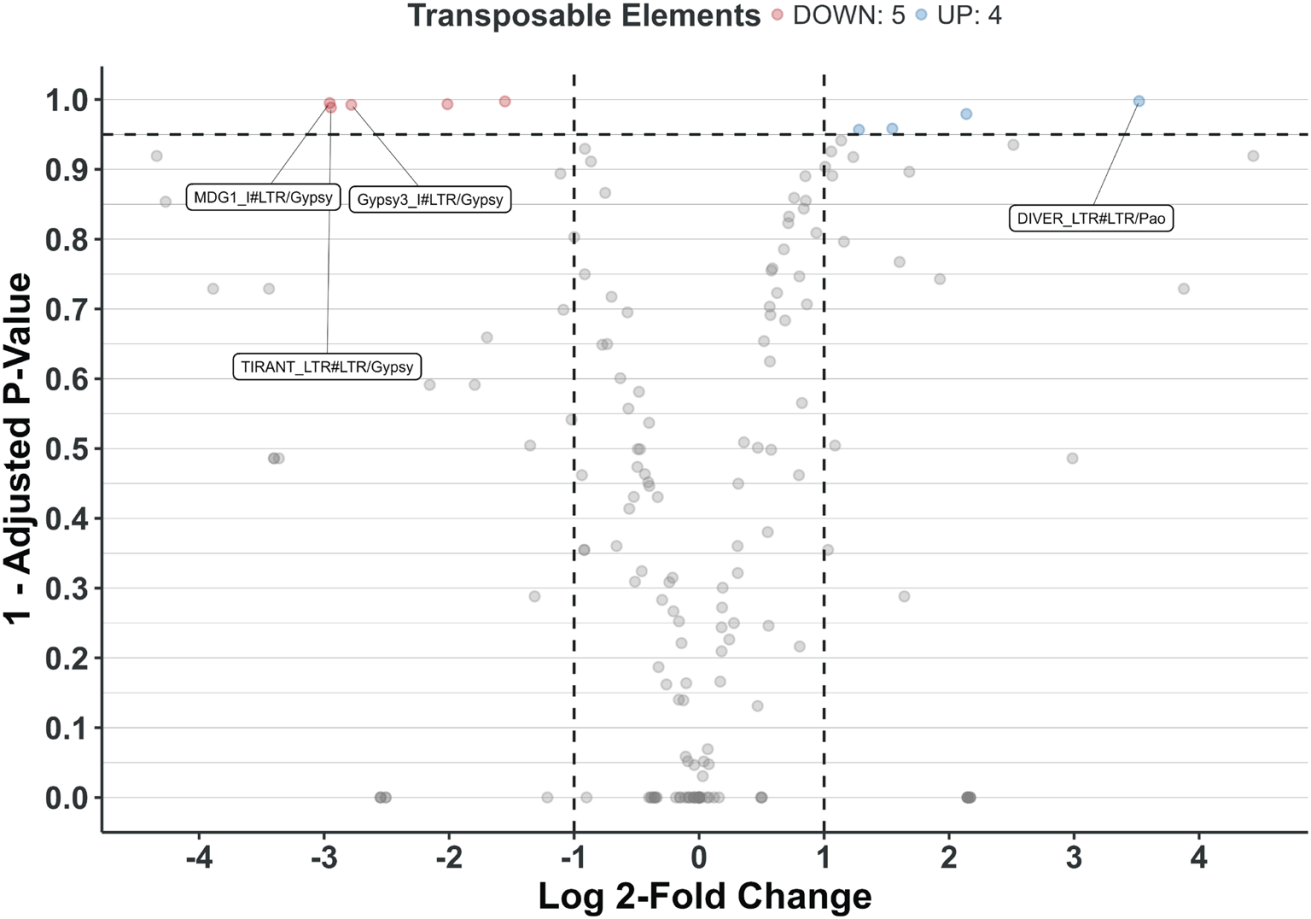
Volcano plot for the transposable elements of the acrylamide‐treated group and control group of *D. melanogaster* transcriptome, by showing variance in gene expression with respect to log_2_foldchange and *p*‐value (adj). Each dot represents an individual gene. Red dots represent the down‐regulated DETEs, Blue dots represent the upregulated DETEs.

To evaluate TE activity in response to acrylamide exposure, TE expression analysis was performed on the transcriptome dataset of the treated and control group using TEspeX. Acrylamide exposure resulted in the transcription of 191 transposable elements. Differential expression analysis, with an adjusted *p*‐value (*p*‐adj) threshold of 0.05 and log_2_ fold change cutoff, identified nine TEs that were significantly expressed (Figure 6). Of the nine differentially expressed TEs, there were two TE subfamilies: *gypsy* and *pao*, with eight belonging to the *gypsy* superfamily (*Gypsy3*, *MDG1_I*, *MDG1*, and *TIRANT*, downregulated; *ZAM_I*, *Gypsy4‐I*, *Invader4_I*, and *TRANSPAC*, upregulated) and one to the *pao* superfamily (*DIVER*, downregulated).

## 4 Discussion

This present study indicates that acrylamide exposure has a distinctive impact on the transcriptomic profile of *D. melanogaster*. Among the 634 differentially expressed genes identified, the majority of the responsive genes were functionally linked to critical biological processes including detoxification pathways, reproduction, metabolism, neurology, enzyme activity, folate metabolism, and oxidoreduction. By focusing on these differentially enriched molecular processes and pathways, we provide a viable molecular foundation for understanding the processes behind the toxic effects of acrylamide‐induced toxicity in *Drosophila*.

The upregulation of genes involved in maltose and glucosidase activities (*Mal‐A2*, *Mal‐A1*, *Mal‐B1*, *Mal‐A7*, *Mal‐A8*, *Mal‐A6*) observed in this study corroborates the hypothesis that acrylamide exposure might be a risk for hyperglycemia and metabolic syndrome [7, 33]. Maltases, classified as α‐glucosidases (EC 3.2.1.20), are crucial in carbohydrate metabolism. Alongside amylase enzymes, maltase participates in starch hydrolysis by hydrolyzing starch into glucose, thereby modulating glucose levels and metabolic pathways [34]. The overexpression of these enzymes suggest that acrylamide exposure might produce postprandial hyperglycemia, through the rapid enzymatic digestion of starch. This rapid hydrolysis could lead to an abrupt increase in glucose level, resulting in impaired insulin secretion over time and contributing to the development of chronic diseases such as obesity and type II diabetes [35]. The downregulation of genes involved in triglyceride lipase activity (*Yp1*, *CG6296*, *CG34447*, *CG14034*, *CG18258*, *CG18301*, *CG31872*, *CG17097*) suggests lipid metabolism dysfunction as another mechanism by which acrylamide may be a risk for metabolic disorder. Reduction in the expression of these enzymes can lead to impaired lipolytic enzymes resulting in unregulated levels of triglycerides. This hypothesis is further supported by the elevated levels of glucose and triglyceride observed in the acrylamide treated group of our study and also in rats [36].

The metabolism of amino acids is fundamental to cellular processes such as protein synthesis, energy production, and cell differentiation. Disruptions in these metabolic pathways can lead to the accumulation of toxic by‐products, thereby contributing to several pathological conditions [37]. This current study observed significant enrichment in the metabolic pathways of amino acids such as tyrosine, arginine, sarcosine, and glycine. These pathways are shown to be associated with key enzymatic functions, including peptidase and serine hydrolase activities and amino acid binding. The differential expression of genes associated with these processes suggests that aberrant amino acid metabolism may play a potential role in acrylamide toxicity.

Functional enrichment analysis of the DE genes revealed several categories that may be contributing to adaptive responses to acrylamide‐induced stress and toxicity: “Toxin metabolic process,” “cellular response to xenobiotics,” “oxidoreductase activity,” “Biological oxidation.” Previous studies have demonstrated that acrylamide exposure results in oxidative stress and biochemical perturbations. Thus, the variance in the transcript expression of multidrug resistance genes (*Mdr 49* and *Mdr 50*), *Nmdmc*, ATP‐binding cassette transporter (ABC), which have physiological roles as transporters for various endogenous compounds and protection against cytotoxic compounds and environmental insults [38, 39] observed in our findings show *Drosophila's* response to the compound and its attempt at detoxification. Enrichment of “heme/tetrapyrolle binding” accounts for another way by which acrylamide can modulate cellular processes. Cytochrome P450s are essential for the detoxification of drugs and other chemicals; however, this can also result in the metabolic activation to yield the reactive metabolites of such parent compounds, initiating toxic events [40]. One of such is the *Cyp6g1* gene, an enzyme that has been revealed to produce metabolites that are toxic but readily excreted. In a study by [40], overexpression of *Cyp6g1* did not lead to the detoxification of imidacloprid, but rather it led to the production of its toxic metabolites. The overexpression of this gene observed in our study may indicate that acrylamide is readily metabolized into its toxic metabolites—glycidamide—and can potentially cause adverse effects. However, the enrichment of the CYP450 family provides further evidence for the toxicity effect of acrylamide.

From our study, acrylamide has a broad, pronounced effect on the expression of genes involved in reproductive process and behavior, an observation in line with other studies [6, 41]. It is interesting to note that over 70% of the downregulated DEGs in our dataset are enriched for GO biological processes associated with “insemination,” “copulation,” “sexual reproduction,” “sperm competition,” and “oviposition.” Studies on internal fertilization, in several species including *Drosophila*, have shown that the transfer of seminal fluid proteins from male to female are crucial for sperm function, storage, release and subsequent post‐mating changes such as female physiology, behavior and refractoriness to mating, defensive and offensive sperm competitiveness, stimulation of egg production, and paternity success. Genetic alteration in the synthesis and secretion of these seminal fluid proteins can cause inadequate receipt and processing of the ejaculate, failure in the formation of mating plugs in mated females, ovulation, egg activation, and overall fertilization [42, 43]. Among the key affected transcripts in our RNA‐seq data are the accessory gland glycoproteins family, seminal fluid protease, ejaculatory bulb protein, serpin family, odorant binding proteins, seminases. Furthermore, the downregulation of Andropin *(Anp)*, a protein expressed in ejaculatory duct, involved in male‐specific antibacterial humoral response, suggests that acrylamide might impact fertility through altered immune modulation. Similarly, the suppressed expression of *CYP4d21*, a circadian‐regulated gene associated with aggression and mating behavior in *D. melanogaster* , shows that acrylamide may modulate male aggressive behavior and disrupt normal mating durations [44]. This potential of acrylamide to perturb circadian‐regulated behaviors aligns with our previous studies, which demonstrated that acrylamide disrupts sleep duration and latency in treated flies [19]. In addition, acrylamide could further comprise fertility by inhibiting sperm‐oviduct binding, due to the distortion in the transcriptional expression of *lectin‐30A* [45]. Together with proposed mechanisms such as oxidative stress, DNA damage, endocrine disruption, and inflammation observed in acrylamide‐treated animals [41, 46], this study was able to corroborate and further highlight that acrylamide can compromise fertility from courtship behavior, sperm quality, modulation of immunity to post‐copulatory events critical to successful fertilization, and reproductive isolation.

The significant down‐expression of lysozyme genes reported in this current study further underscores the impact of acrylamide on immune modulation. Research studies highlight lysozyme as a cornerstone of innate immunity and a key modulator of host immune response to infection [47]. This aligns with previous studies reporting acrylamide‐induced immunotoxicity through alternative pathways [48]. Our ontology analysis also revealed enrichment of biological processes associated with folate‐dependent one‐carbon metabolism was also present in our ontology analysis. This includes “one‐carbon metabolic process” “tetrahydrofolate interconversion” “tetrahydrofolate metabolic process.” Folate‐dependent one‐carbon metabolism accounts for a complex network of biochemical reactions essential for maintaining cellular redox balance, biosynthesis of crucial molecules, energy production, and amino acid metabolism. One of the highlighted roles of this process is the epigenetic regulation of DNA and RNA methylation [49]. This process facilitates epigenetic regulation of gene expression through the production of S‐adenosylmethionine (SAM), the universal methyl donor required by most methyltransferases for DNA and histones methylation [50]. Dysregulation of genes involved in this pathway recommends alternative pathways by which acrylamide can affect cellular processes and induce histone epigenetic alterations.

One of the significant adverse health effects of acrylamide is neurotoxicity, with its neurotoxic effect at both short and long‐term exposure well‐demonstrated in both humans and animals [4, 51, 52]. One of such is a case that involved contaminated well water of up to 400 mg/L acrylamide causing memory disturbances, hallucinations, confusion in exposed individuals. More severe exposure to a potentially lethal dose of 500 mg/kg/body weight, manifested pronounced neurotoxic symptoms including ataxia, resting tremors and reduced deep tendon reflexes [53]. Findings from this study provide further clarification for the mechanisms of acrylamide‐induced neurotoxicity. Membrane potential, which represents electrical potential difference between intracellular and extracellular environments, is tightly controlled by ion channels and pumps [54]. The increase in the “regulation of membrane potential” biological process observed in this study highlights a potential mechanism underlying acrylamide‐induced neurotoxicity. Specifically, acrylamide overexpresses voltage‐gated sodium (Na^+^) and calcium (Ca^2+^) channels, promoting increased influx of these cations thereby inducing depolarization. Our results further indicate that acrylamide can modulate ion channel function by enhancing the activities of ion channel regulators, *tipE Rgk1*, *inaF‐C*, *inaF‐B* as well as ligand‐gated ions channels (*ppk12*, *Ir75a*, *ppk3*, *ppk8*, *CngB*, *Grd*, *TrpA*, *nAChRbeta2*). This dysregulation may contribute to denervation super‐sensitivity or hyperactivation sub‐sensitivity across several neurotransmitter systems. Thus, the alterations in intracellular Ca^2+^ and Na^+^ levels can serve as a crucial biomarker for the investigation of the effects of acrylamide on neuronal network activity. Moreover, the activation of nociceptor cation channel *TRPA1* and its subunits *INAF‐C* and *INAF‐B* suggests an additional mechanistic pathway through which acrylamide exerts one of its downstream effects, inflammation. *INAF‐B* and *INAF‐C* are core components of the signalplex, a multiprotein array that regulates the activity of *TRPA1* [55]. Several studies have demonstrated the involvement of *TRPA1* in pro‐inflammatory responses upon its activation by endogenous and exogenous irritants [56, 57, 58]. *TRPA1* can trigger neurogenic inflammatory response by coordinating a series of cellular processes, including production of cytokines release of neuropeptides and an increase in Ca^2+^ influx [59]. This pathway may also be implicated in acrylamide‐induced respiratory diseases as *TRPA1* plays a key role in reactive airway diseases and airway chemosensation of noxious tobacco smoke constituents [56]. Additionally, *TRPA1* channel may modulate social and emotional behaviors. Notably, overexpression of *TRPA1* in mice contributed to avoidance behavior, loss of learning and memory [60]. This suggests that *TRPA1* upregulation is a plausible pathway through which acrylamide could influence behavioral changes as observed in our previous study [19] and memory deficit observed in this current study.

Beyond the above stated mechanism of acrylamide impact on synapse signaling and transmission, acrylamide also disrupts pathways governing synapse organization and formation through the overexpression of associated genes such as *Wnt4*, *beat‐lllc*, and *Appl*. This is consistent with previous studies that reported that the ectopic expression of *Wnt4* and *beat‐lllc* inhibits synaptogenesis [61], while *Appl* overexpression markedly increased synaptic bouton number and changes in synapse structure [62]. The possibility of acrylamide to induce neurodegeneration through structural remodeling was also reported by Bin‐Jumah et al. [4], where ultrastructural alterations such as changes in the size and positioning of Nissl bodies and mitochondria were observed in the nervous system of albino rats exposed to acrylamide (50 mg/kg/day). Acrylamide exposure has been implicated in the early onset of neurodegenerative disorders such as cognitive decline, Alzheimer's disease, Parkinson disease, and dementia [63], a theory supported by the detection of elevated expression levels of genes such as *Tau*, *Appl*, *Ddc*, *DopEcR*. Consistently, the significant reduction in the performance index observed during the negative association memory test in exposed flies suggests a direct impact on short‐term memory. Given this performance decline, we further examined the transcriptome for genes annotated as “learning or memory” in the FlyBase dataset. Sixteen genes (Table 2) were identified as strong candidates, potentially underlying memory deficits. Of note is the *NMDA receptor 1 (Nmdar1)* gene, highlighted as a hub within the protein–protein interaction network (Figure 4b). Prior studies have shown that sub‐acute exposure of benzo(a) pyrene in adult mice impairs short‐term and spatial memory through increased *Nmdar1* expression [64], and *NMDA* receptor function is known to be regulated by the amyloid precursor protein, with age‐related increases in *APP* and *NMDA* receptor resulting in aberrant *NMDA* receptor function and synaptic dysfunctions [65]. Our observation of concurrent increases in *Appl* and *Nmdar1* suggests that acrylamide may induce memory deficit and cognitive decline through similar mechanisms. Plus, the downregulation of *Milkah (mil)*, which is implicated in long‐term memory retention in *Drosophila* [66], hints at potential impacts on term memory. The elevated expression of *DopEcR* and *Ddc* further implicates dysregulated dopaminergic signaling in acrylamide‐induced memory and learning deficits.

Several studies have demonstrated that stressors can trigger the mobilization of transposable elements (TEs). Various stressors such as heavy metals, cyclophosphamide and cisplatin have been shown to induce TE expression across diverse organisms such as plants, rodents, cell lines, humans and *D. melanogaster* [15, 16, 67]. In our study, we identified nine differentially expressed TEs (DETEs) belonging to the Gypsy/Pao subfamilies, a group within the long terminal repeat (LTR) retrotransposon class. Among these, four were downregulated and five upregulated. While there is ongoing debate as to whether TE activation results from their proximity to stress‐induced genes or it is a direct consequence of stress exposure [14], we hypothesize that TE activation is directly induced by acrylamide exposure and its associated toxicity. Although transcription represents only the initial step toward mobilization, increased TE transcription is generally associated with enhanced mobility [15]. TE‐derived transcripts have diverse functional implications. In most cases, these TE‐derived transcripts may result in partial transcripts that lack full coding potential for transposition‐related proteins, but sometimes they may also encode RNA and proteins essential for transposition. Also, these TE‐derived RNAs can also result in small RNAs that might influence silencing of other related TEs or genes, or act as outward‐facing promoters that modify the expression or splicing of nearby protein‐coding genes [68]. Thus, TE's overexpression observed in this current study may influence gene regulatory networks involved in detoxification, oxidation stress responses or genomic instability, while the downregulation might signify the restoration of silence to prevent excessive genomic perturbation. However, whether acrylamide‐induced TE expression results from disrupted control mechanisms and if these TEs exert biological consequences upon expression remains unknown at this moment. More studies are needed to investigate the responses of TEs to different environmental pollutants and their possible downstream effect. This would provide mechanistic clarity as to whether TEs are drivers of their induced toxicity or just secondary responders to cellular stress.

## 5 Conclusion

Our study provides a comprehensive transcriptomic analysis of acrylamide exposure in *D. melanogaster* using RNA‐Seq technology. Differentially expressed genes (DEGs) were enriched in pathways critical to metabolism, immunity, epigenetic regulation, reproduction, detoxification, folate biosynthesis, and neurotransmission. To the best of our knowledge, this study is the first to report that acrylamide exposure promotes transposable elements' differential expression, highlighting novel pathways by which acrylamide exerts its toxicity. These findings substantially contribute to our understanding of the mechanistic pathway underlying acrylamide toxicity and provide a framework for future research. Future investigations should prioritize long‐term environmentally relevant dose exposure via various routes of human exposure in order to assess the multi‐synergistic effect of acrylamide and identify new candidate biomarkers that could enhance human biomonitoring strategies and early detection of acrylamide‐related health risks.

## Author Contributions

**Oluwabukola Mary Farodoye:** writing – review and editing, writing – original draft, validation, investigation, formal analysis, conceptualization. **Titilayomi Ayomide Otenaike:** writing – original draft, validation, methodology, investigation. **Daniela Moreira Mombach:** writing – original draft, bioinformatics, investigation. **Monica Medeiros Silva:** methodology, data curation. **Amos Olalekan Abolaji:** writing – original draft, conceptualization. **Elgion Lucio Da Silva Loreto:** writing – review and editing, writing – original draft, validation, supervision, project administration, funding acquisition, data curation, conceptualization.

## Funding

This work was supported by the Conselho Nacional de Desenvolvimento Científico e Tecnológico (305613/2020‐0).

## Conflicts of Interest

The authors declare no conflicts of interest.

## Supporting information


**Table S1a:** List of up‐regulated genes and their enriched molecular functions, including the pathway and their gene ontology ID.
**Table S1a:** List of Down‐regulated genes and their enriched molecular functions, including the pathway and their gene ontology ID.

**Table S2a:** List of up‐regulated genes and their enriched biological processes with pathways and gene ontology ID.
**Table S2b:** Down‐regulated genes and their enriched biological processes with pathways and gene ontology ID.

**Table S3:** Differentially regulated REACTOME pathways in acrylamide‐treated *D. melanogaster* compared with the control. Pathway enrichment analysis was based on Bonferroni‐corrected *p* < 0.05.

**Table S4:** All differentially expressed genes marked as “Learning or Memory” in FlyBase dataset with expression values in Transcripts per Million (TPM) and Fold Change annotated. Negative values signify down regulated while positive values signify upregulation.

## Data Availability

Data supporting the findings reported in this study are available upon reasonable reques.
