# Supplementary material for: Diving Deeper Into Mechanisms of Acrylamide‐Induced Toxicity: RNA Sequencing Reveals Transcriptomic Alteration and Retrotransposon Expression in Drosophila melanogaster
Source: Environ Toxicol. 2026 Feb 23;41(10):1105–18. doi: 10.1002/tox.70054 (PMC13537343; doi:10.1002/tox.70054)
Supplement: Supplementary file 1 [file TOX-41-1105-s001.docx]

Supplementary Table 3: Differentially regulated REACTOME pathways in acrylamide-treated *D. melanogaster* compared with the control. Pathway enrichment analysis was based on Bonferroni-corrected p < 0.05.

| **S/N** | **REACTOME Pathway Term/ID** | **No of genes** | **PValue** | **Fold Enrichment** |
| --- | --- | --- | --- | --- |
| **1** | R-DME-556833~Metabolism of lipids | 38 | 6.25E-08 | 2.558009 |
| **2** | R-DME-1430728~Metabolism | 66 | 8.09E-07 | 1.715594 |
| **3** | R-DME-9748784~Drug ADME | 17 | 4.32E-06 | 3.936317 |
| **4** | R-DME-500792~GPCR ligand binding | 12 | 8.65E-06 | 5.441379 |
| **5** | R-DME-373076~Class A/1 (Rhodopsin-like receptors) | 11 | 1.59E-05 | 5.700493 |
| **6** | R-DME-196071~Metabolism of steroid hormones | 11 | 2.12E-05 | 5.525093 |
| **7** | R-DME-425407~SLC-mediated transmembrane transport | 19 | 1.19E-04 | 2.781692 |
| **8** | R-DME-382551~Transport of small molecules | 32 | 1.31E-04 | 2.020783 |
| **9** | R-DME-8978868~Fatty acid metabolism | 14 | 4.06E-04 | 3.152247 |
| **10** | R-DME-8957322~Metabolism of steroids | 12 | 4.53E-04 | 3.56163 |
| **11** | R-DME-112310~Neurotransmitter release cycle | 9 | 4.54E-04 | 4.816959 |
| **12** | R-DME-352230~Amino acid transport across the plasma membrane | 8 | 6.13E-04 | 5.330331 |
| **13** | R-DME-112316~Neuronal System | 16 | 0.001867 | 2.452453 |
| **14** | R-DME-2142753~Arachidonate metabolism | 6 | 0.001873 | 6.529655 |
| **15** | R-DME-372790~Signaling by GPCR | 14 | 0.003016 | 2.53931 |
| **16** | R-DME-1483206~Glycerophospholipid biosynthesis | 11 | 0.0031 | 3.017908 |
| **17** | R-DME-381426~Regulation of Insulin-like Growth Factor (IGF) transport and uptake by Insulin-like Growth Factor Binding Proteins (IGFBPs) | 8 | 0.003614 | 3.957367 |
| **18** | R-DME-8957275~Post-translational protein phosphorylation | 8 | 0.003614 | 3.957367 |
| **19** | R-DME-9757110~Prednisone ADME | 8 | 0.003935 | 3.898302 |
| **20** | R-DME-193144~Estrogen biosynthesis | 6 | 0.004281 | 5.441379 |
| **21** | R-DME-8935690~Digestion | 7 | 0.004587 | 4.39496 |
| **22** | R-DME-1482801~Acyl chain remodelling of PS | 6 | 0.005429 | 5.154991 |
| **23** | R-DME-112315~Transmission across Chemical Synapses | 13 | 0.005731 | 2.467602 |
| **24** | R-DME-388396~GPCR downstream signalling | 13 | 0.006279 | 2.439239 |
| **25** | R-DME-425366~Transport of bile salts and organic acids, metal ions and amine compounds | 8 | 0.006339 | 3.577893 |
| **26** | R-DME-8963743~Digestion and absorption | 7 | 0.006624 | 4.081034 |
| **27** | R-DME-416476~G alpha (q) signalling events | 9 | 0.00693 | 3.159511 |
| **28** | R-DME-211976~Endogenous sterols | 5 | 0.007289 | 6.278515 |
| **29** | R-DME-181430~Norepinephrine Neurotransmitter Release Cycle | 6 | 0.008349 | 4.664039 |
| **30** | R-DME-418555~G alpha (s) signalling events | 6 | 0.008349 | 4.664039 |
| **31** | R-DME-211897~Cytochrome P450 - arranged by substrate type | 5 | 0.009525 | 5.830049 |
| **32** | R-DME-5653890~Lactose synthesis | 4 | 0.009587 | 8.706207 |
| **33** | R-DME-264642~Acetylcholine Neurotransmitter Release Cycle | 4 | 0.01154 | 8.162069 |
| **34** | R-DME-181429~Serotonin Neurotransmitter Release Cycle | 4 | 0.01154 | 8.162069 |
| **35** | R-DME-8963889~Assembly of active LPL and LIPC lipase complexes | 5 | 0.013644 | 5.265851 |
| **36** | R-DME-375276~Peptide ligand-binding receptors | 5 | 0.013644 | 5.265851 |
| **37** | R-DME-211859~Biological oxidations | 9 | 0.016375 | 2.72069 |
| **38** | R-DME-174824~Plasma lipoprotein assembly, remodeling, and clearance | 7 | 0.0166 | 3.360852 |
| **39** | R-DME-9753281~Paracetamol ADME | 6 | 0.01714 | 3.917793 |
| **40** | R-DME-212676~Dopamine Neurotransmitter Release Cycle | 4 | 0.018685 | 6.873321 |
| **41** | R-DME-192456~Digestion of dietary lipid | 5 | 0.018751 | 4.801217 |
| **42** | R-DME-1483257~Phospholipid metabolism | 11 | 0.018895 | 2.316974 |
| **43** | R-DME-114608~Platelet degranulation | 9 | 0.019038 | 2.647158 |
| **44** | R-DME-76005~Response to elevated platelet cytosolic Ca2+ | 9 | 0.021998 | 2.577495 |
| **45** | R-DME-425393~Transport of inorganic cations/anions and amino acids/oligopeptides | 9 | 0.026435 | 2.490123 |
| **46** | R-DME-8964058~HDL remodeling | 5 | 0.032144 | 4.081034 |
| **47** | R-DME-9845614~Sphingolipid catabolism | 3 | 0.035232 | 9.794483 |
| **48** | R-DME-140837~Intrinsic Pathway of Fibrin Clot Formation | 4 | 0.038865 | 5.223724 |
| **49** | R-DME-140875~Common Pathway of Fibrin Clot Formation | 4 | 0.038865 | 5.223724 |
| **50** | R-DME-140877~Formation of Fibrin Clot (Clotting Cascade) | 4 | 0.038865 | 5.223724 |
| **51** | R-DME-2187338~Visual phototransduction | 5 | 0.040498 | 3.796311 |
| **52** | R-DME-3000178~ECM proteoglycans | 4 | 0.042984 | 5.022812 |
| **53** | R-DME-8963899~Plasma lipoprotein remodeling | 5 | 0.043532 | 3.710031 |
| **54** | R-DME-9709957~Sensory Perception | 5 | 0.043532 | 3.710031 |
| **55** | R-DME-194002~Glucocorticoid biosynthesis | 4 | 0.047312 | 4.836782 |
| **56** | R-DME-5423646~Aflatoxin activation and detoxification | 3 | 0.049669 | 8.162069 |
