# Supplementary material for: Diving Deeper Into Mechanisms of Acrylamide‐Induced Toxicity: RNA Sequencing Reveals Transcriptomic Alteration and Retrotransposon Expression in Drosophila melanogaster
Source: Environ Toxicol. 2026 Feb 23;41(10):1105–18. doi: 10.1002/tox.70054 (PMC13537343; doi:10.1002/tox.70054)
Supplement: Supplementary file 2 [file TOX-41-1105-s002.docx]

SUPPLEMENTARY TABLE 4: All differentially expressed genes marked as “Learning or Memory” in FlyBase dataset with expression values in Transcripts per Million (TPM) and Fold Change annotated. Negative values signify down regulated while positive values signify upregulation.

| **FlyBaseID** | **GeneName** | **Control**  **(TPM Mean)** | **Acrylamide**  **(TPM Mean)** | | **Fold**  **Change** |
| --- | --- | --- | --- | --- | --- |
| FBgn0016919 | *no mechanoreceptor potential B(nompB)* | 483.17 | | 179.32 | -2.69444 |
| FBgn0267366 | *milkah(mil)* | 959.73 | | 372.61 | -2.57571 |
| FBgn0051148 | *Glucocerebrosidase 1a(Gba1a)* | 25,577.37 | | 11,329.78 | -2.25754 |
| FBgn0028396 | *Turandot A(TotA)* | 740,432.72 | | 331,537.45 | -2.23333 |
| FBgn0039270 | *Poly-glutamine tract binding protein 1(PQBP1)* | 16,496.81 | | 9,306.50 | -1.77261 |
| FBgn0034443 | *crammer(cer)* | 14,199.22 | | 8,904.68 | -1.59458 |
| FBgn0086782 | *amnesiac(amn)* | 291.75 | | 195.68 | -1.49096 |
| FBgn0262124 | *unextended(uex)* | 12,672.50 | | 8,856.01 | -1.43095 |
| FBgn0011582 | *Dopamine 1-like receptor 1(Dop1R1)* | 147.00 | | 103.20 | -1.42442 |
| FBgn0042185 | *Mitochondrial calcium uniporter(MCU)* | 7,083.76 | | 5,091.82 | -1.3912 |
| FBgn0004419 | *maternal expression at 31B(me31B)* | 369,450.74 | | 271,195.50 | -1.3623 |
| FBgn0037913 | *fatty acid binding protein(fabp)* | 132,599.14 | | 103,518.24 | -1.28093 |
| FBgn0029114 | *tollo(Tollo)* | 3,509.26 | | 2,743.95 | -1.27891 |
| FBgn0040372 | *G9a(G9a)* | 18,743.63 | | 14,940.31 | -1.25457 |
| FBgn0043002 | *Chromatin accessibility complex 14kD protein(Chrac-14)* | 51,091.20 | | 41,261.89 | -1.23822 |
| FBgn0046114 | *Glutamate-cysteine ligase modifier subunit(Gclm)* | 194,968.34 | | 171,213.16 | -1.13875 |
| FBgn0011768 | *Formaldehyde dehydrogenase(Fdh)* | 222,405.11 | | 196,835.79 | -1.1299 |
| FBgn0004648 | *silver(svr)* | 10,358.64 | | 12,129.97 | 1.171001 |
| FBgn0266557 | *kismet(kis)* | 10,821.93 | | 12,696.00 | 1.173174 |
| FBgn0259481 | *Mob2(Mob2)* | 33,909.01 | | 39,828.56 | 1.174572 |
| FBgn0003520 | *staufen(stau)* | 19,223.93 | | 22,661.60 | 1.178822 |
| FBgn0000119 | *arrow(arr)* | 5,736.91 | | 6,852.10 | 1.194389 |
| FBgn0000546 | *Ecdysone receptor(EcR)* | 4,602.37 | | 5,500.52 | 1.195151 |
| FBgn0014141 | *cheerio(cher)* | 30,425.03 | | 36,551.21 | 1.201353 |
| FBgn0010762 | *simjang(simj)* | 16,379.56 | | 19,746.38 | 1.20555 |
| FBgn0261673 | *no extended memory(nemy)* | 13,916.35 | | 16,794.35 | 1.206807 |
| FBgn0024944 | *Octopamine receptor in mushroom bodies(Oamb)* | 1,844.49 | | 2,257.43 | 1.223877 |
| FBgn0013759 | *CASK(CASK)* | 12,064.00 | | 14,773.03 | 1.224555 |
| FBgn0261854 | *atypical protein kinase C(aPKC)* | 13,145.77 | | 16,124.49 | 1.226592 |
| FBgn0041210 | *Histone deacetylase 4(HDAC4)* | 11,927.15 | | 14,860.40 | 1.245931 |
| FBgn0265784 | *Cyclic-AMP response element binding protein B(CrebB)* | 16,882.40 | | 21,113.33 | 1.250611 |
| FBgn0000229 | *basket(bsk)* | 14,763.70 | | 18,562.13 | 1.257281 |
| FBgn0000017 | *Abl tyrosine kinase(Abl)* | 5,210.27 | | 6,607.99 | 1.268262 |
| FBgn0026401 | *Nipped-B(Nipped-B)* | 4,546.14 | | 5,768.23 | 1.268819 |
| FBgn0264607 | *Calcium/calmodulin-dependent protein kinase II(CaMKII)* | 10,003.66 | | 12,768.19 | 1.276352 |
| FBgn0022382 | *Protein kinase, cAMP-dependent, regulatory subunit type 2(Pka-R2)* | 13,439.64 | | 17,317.17 | 1.288515 |
| FBgn0003984 | *vein(vn)* | 699.03 | | 909.35 | 1.300871 |
| FBgn0051414 | *Glucocerebrosidase 1b(Gba1b)* | 4,642.20 | | 6,077.65 | 1.309218 |
| FBgn0053207 | *pxb(pxb)* | 1,120.16 | | 1,484.60 | 1.325355 |
| FBgn0052062 | *RNA-binding Fox protein 1(Rbfox1)* | 5,648.91 | | 7,501.92 | 1.328029 |
| FBgn0001208 | *Henna(Hn)* | 62,169.96 | | 82,790.45 | 1.331679 |
| FBgn0003392 | *shibire(shi)* | 21,599.56 | | 29,105.61 | 1.34751 |
| FBgn0010015 | *Calcineurin A1(CanA1)* | 2,952.55 | | 4,055.06 | 1.37341 |
| FBgn0005672 | *spitz(spi)* | 18,033.14 | | 24,928.39 | 1.382366 |
| FBgn0003371 | *shaggy(sgg)* | 10,366.14 | | 14,375.09 | 1.386736 |
| FBgn0036896 | *wallenda(wnd)* | 5,243.16 | | 7,367.92 | 1.405244 |
| FBgn0003861 | *transient receptor potential(trp)* | 48,056.20 | | 67,651.16 | 1.407751 |
| FBgn0031603 | *uncharacterized protein(CG15432)* | 19,735.26 | | 28,019.63 | 1.419775 |
| FBgn0000635 | *Fasciclin 2(Fas2)* | 15,993.18 | | 22,815.29 | 1.426563 |
| FBgn0013334 | *Synapse-associated protein 47kD(Sap47)* | 13,569.62 | | 19,413.87 | 1.430686 |
| FBgn0003996 | *white(w)* | 12,230.36 | | 17,602.31 | 1.439231 |
| FBgn0003093 | *Protein C kinase 98E(Pkc98E)* | 9,841.10 | | 14,641.52 | 1.487794 |
| FBgn0261788 | *Ankyrin 2(Ank2)* | 1,433.10 | | 2,169.29 | 1.513702 |
| FBgn0262739 | *Argonaute-1(AGO1)* | 12,869.42 | | 19,639.25 | 1.52604 |
| FBgn0030600 | *highwire(hiw)* | 2,235.85 | | 3,464.97 | 1.54973 |
| FBgn0039747 | *Adenosine receptor(AdoR)* | 314.15 | | 489.73 | 1.558894 |
| FBgn0037140 | *SLC22A family member(SLC22A)* | 6,385.17 | | 10,153.65 | 1.590192 |
| FBgn0003068 | *period(per)* | 6,608.09 | | 10,876.26 | 1.6459 |
| FBgn0259108 | *futsch(futsch)* | 932.06 | | 1,562.61 | 1.676522 |
| FBgn0023023 | *Collapsin Response Mediator Protein(CRMP)* | 6,948.69 | | 11,854.26 | 1.705971 |
| FBgn0019985 | *metabotropic Glutamate Receptor(mGluR)* | 421.12 | | 723.85 | 1.718888 |
| FBgn0259246 | *bruchpilot(brp)* | 672.46 | | 1,158.99 | 1.723512 |
| FBgn0260793 | *2mit(2mit)* | 468.94 | | 809.91 | 1.727109 |
| FBgn0041111 | *lilliputian(lilli)* | 4,586.28 | | 8,009.41 | 1.746384 |
| FBgn0266098 | *rugose(rg)* | 51.82 | | 90.87 | 1.753526 |
| FBgn0037703 | *Lysine demethylase 3(Kdm3)* | 1,992.04 | | 3,508.00 | 1.761007 |
| FBgn0004244 | *Resistant to dieldrin(Rdl)* | 1,171.05 | | 2,079.26 | 1.775546 |
| FBgn0031888 | *PDGF- and VEGF-related factor 2(Pvf2)* | 1,965.09 | | 3,543.98 | 1.803467 |
| FBgn0053513 | *NMDA receptor 2(Nmdar2)* | 322.78 | | 589.03 | 1.824867 |
| FBgn0284084 | *wingless(wg)* | 1,316.67 | | 2,446.71 | 1.858256 |
| FBgn0004168 | *5-hydroxytryptamine (serotonin) receptor 1A(5-HT1A)* | 321.14 | | 612.10 | 1.906039 |
| FBgn0040505 | *Anaplastic lymphoma kinase(Alk)* | 1,341.35 | | 2,629.77 | 1.960544 |
| FBgn0001123 | *G protein alpha subunit s(Galphas)* | 9,830.54 | | 19,394.52 | 1.972885 |
| FBgn0038975 | *Neurexin 1(Nrx-1)* | 1,822.45 | | 3,925.06 | 2.153726 |
| FBgn0010329 | *Tyramine beta hydroxylase(Tbh)* | 774.86 | | 1,688.47 | 2.179074 |
| FBgn0005564 | *Shaker cognate l(Shal)* | 1,782.52 | | 3,888.27 | 2.181327 |
| FBgn0000108 | *beta amyloid protein precursor-like(Appl)* | 3,800.52 | | 8,728.00 | 2.296526 |
| FBgn0004575 | *Synapsin(Syn)* | 1,361.92 | | 3,177.15 | 2.332847 |
| FBgn0010399 | *NMDA receptor 1(Nmdar1)* | 444.55 | | 1,042.37 | 2.34479 |
| FBgn0004516 | *Glutamic acid decarboxylase 1(Gad1)* | 7,081.17 | | 18,244.43 | 2.57647 |
| FBgn0035538 | *Dopamine/Ecdysteroid receptor(DopEcR)* | 1,929.87 | | 5,061.76 | 2.622856 |
| FBgn0017590 | *klingon(klg)* | 1,248.15 | | 3,314.68 | 2.655681 |
| FBgn0000422 | *Dopa decarboxylase(Ddc)* | 6,796.64 | | 18,357.49 | 2.700965 |
| FBgn0000535 | *ether a go-go(eag)* | 232.03 | | 664.88 | 2.865447 |
| FBgn0264753 | *Rad, Gem/Kir family member 1(Rgk1)* | 573.48 | | 1,747.38 | 3.046992 |
| FBgn0031854 | *Tubulin tyrosine ligase-like 3A(TTLL3A)* | 152.27 | | 517.24 | 3.396768 |
| FBgn0004956 | *unpaired 1(upd1)* | 282.11 | | 1,049.17 | 3.71901 |
| FBgn0028513 | *uncharacterized protein(CG9254)* | 224.36 | | 970.90 | 4.327375 |
| FBgn0262477 | *Forkhead box P(FoxP)* | 57.36 | | 290.26 | 5.060074 |
| FBgn0028740 | *uncharacterized protein(CG6362)* | 134.34 | | 1,504.85 | 11.20199 |
