# Supplementary material for: Diving Deeper Into Mechanisms of Acrylamide‐Induced Toxicity: RNA Sequencing Reveals Transcriptomic Alteration and Retrotransposon Expression in Drosophila melanogaster
Source: Environ Toxicol. 2026 Feb 23;41(10):1105–18. doi: 10.1002/tox.70054 (PMC13537343; doi:10.1002/tox.70054)
Supplement: Supplementary file 3 [file TOX-41-1105-s003.docx]

**Supplementary Table 2a: List of Up-regulated genes and their enriched biological processes with pathways and gene ontology ID**

| **Pathway** | **Genes** | **Fold Enrichment** |
| --- | --- | --- |
| GO:1901052 sarcosine metabolic process | Sardh ,Gnmt | 28.48 |
| GO:0000023 maltose metabolic process | Mal-A2, Mal-A1, Mal-B1, Mal-A7, Mal-A8, Mal-A6 | 25.63 |
| GO:0046680 response to DDT | Cyp6g1, Cyp6w1, Cyp6g2, Cyp12d1-p, Cyp12d1-d | 23.73 |
| GO:0009407 toxin catabolic process | Cyp6g1, Cyp6w1, Cyp6g2 | 21.36 |
| GO:0042178 xenobiotic catabolic process | Cyp6g1, Cyp6w1, Cyp6g2 | 21.36 |
| GO:0042908 xenobiotic transport | CG10226, Mdr49, Mdr50 | 21.36 |
| GO:0046701 insecticide catabolic process | Cyp6g1, Cyp6w1, Cyp6g2 | 21.36 |
| GO:0009404 toxin metabolic process | Cyp6g1, Cyp6w1, Cyp6g2 | 18.31 |
| GO:0017143 insecticide metabolic process | Cyp6g1, Cyp6w1, Cyp6g2 | 18.31 |
| GO:0007190 activation of adenylate cyclase activity | Lgr1, Lgr4 | 17.09 |
| GO:0007263 nitric oxide mediated signal transduction | Nos, Gyc88E | 17.09 |
| GO:0010378 temperature compensation of the circadian clock | eys, TrpA1 | 17.09 |
| GO:0035999 tetrahydrofolate interconversion | Nmdmc, pug | 17.09 |
| GO:0017085 response to insecticide | Cyp6g1, Cyp6w1, Cyp6g2, nAChRbeta2, Mdr49, Mdr50, Cyp12d1-p, Cyp12d1-d | 16.27 |
| GO:0046653 tetrahydrofolate metabolic process | Nmdmc, pug, CG8665 | 16.02 |
| GO:0097254 renal tubular secretion | Mdr49, Mdr50, CG10226 | 14.24 |
| GO:0005984 disaccharide metabolic process | Mal-A2, Mal-A1, Mal-B1, Mal-A7, Mal-A8, Mal-A6 | 12.82 |
| GO:0007588 excretion | Mdr49, Mdr50, CG10226 | 12.82 |
| GO:0031281 positive regulation of cyclase activity | Nos, Lgr1, Lgr4 | 12.82 |
| GO:0051349 positive regulation of lyase activity | Nos, Lgr1, Lgr4 | 12.82 |
| GO:0006805 xenobiotic metabolic process | Cyp6g1, Cyp6w1, Cyp6g2, Ugt36F1 | 10.68 |
| GO:0071466 cellular response to xenobiotic stimulus | Cyp6g1, Cyp6w1, Cyp6g2, Ugt36F1 | 10.68 |
| GO:0006760 folic acid-containing compound metabolic process | Nmdmc, pug, CG8665 | 10.68 |
| GO:0031279 regulation of cyclase activity | Nos, Lgr1, Lgr4 | 9.86 |
| GO:0051339 regulation of lyase activity | Nos, Lgr1, Lgr4 | 9.86 |
| GO:0042219 cellular modified amino acid catabolic process | CG17636, Sardh, CG8665 | 9.15 |
| GO:0006570 tyrosine metabolic process | Hpd, yellow-h, Ddc | 8.54 |
| GO:0016201 synaptic target inhibition | robo3, Wnt4, beat-IIIc | 8.54 |
| GO:0009410 response to xenobiotic stimulus | Cyp6g1, Cyp6w1, Cyp6g2, Ugt36F1 | 8.14 |
| GO:0007638 mechanosensory behavior | TrpA1, jus, Tmc | 7.54 |
| GO:0009311 oligosaccharide metabolic process | Mal-A2, Mal-A1, Mal-B1, Mal-A7, Mal-A8, Mal-A6 | 6.57 |
| GO:0006730 one-carbon metabolic process | Nmdmc, pug, Gnmt, CG8665 | 5.34 |
| GO:1901606 alpha-amino acid catabolic process | Nos, Sardh, Hpd, CG5955, CG3999, Gad1 | 4.49 |
| GO:0045055 regulated exocytosis | Snap25, cpx, CG31475, Cadps, Syn, Rph, Syngr | 4.39 |
| GO:0007218 neuropeptide signaling pathway | CG13579, AstA-R2, NPF, Hug CCHa2-R, Lgr1, Nplp1, Lgr4 | 4.38 |
| GO:0009636 response to toxic substance | Cyp6g1, Cyp6w1, Cyp6g2, GstD8, CG33177, Mdr49, Mdr50, CG10226, nAChRbeta2, Zip71B, Cyp12d1-p, Cyp12d1 | 4.27 |
| GO:0042391 regulation of membrane potential | Grd, nAChRbeta2, Ir75a, CG42340, Ca-alpha1T, jus, shakB, inaF-B, inaF-C | 4.09 |
| GO:0016079 synaptic vesicle exocytosis | Snap25, cpx, Cadps, Syn, Rph, Syngr | 4.07 |
| GO:0006575 cellular modified amino acid metabolic process | GstD8, Nmdmc, pug, CG17636, Sardh, GstD9, Gnmt, CG8665 | 3.76 |
| GO:1901605 alpha-amino acid metabolic process | Nos, Sardh, Hpd, CG5955, CG3999, Gnmt, yellow-h, pug, Ddc, Gad1 | 3.71 |
| GO:0014070 response to organic cyclic compound | Cyp6g1, Cyp6w1, Cyp6g2, DopEcR, 5-HT1B, ph-p, Cyp12d1-p, Cyp12d1-d, Kr-h1 | 3.31 |
| GO:0099504 synaptic vesicle cycle | Snap25, cpx, Nlg4, Syngr, Cadps, bw, Syn, Rph | 2.97 |
| GO:0099003 vesicle-mediated transport in synapse | Snap25, cpx, Nlg4, Syngr, Cadps, bw, Syn, Rph | 2.94 |
| GO:0016054 organic acid catabolic process | Nos, Sardh, Hpd, CG5955, CG3999, CG8665, Gad1, CG3699 | 2.89 |
| GO:0006520 cellular amino acid metabolic process | Nos, Sardh, Hpd, CG5955, CG3999, Gnmt, yellow-h, Ddc, pug, CG6465, Gad1 | 2.67 |
| GO:0006790 sulfur compound metabolic process | GstD8, CG17636, Sgp, CG12913, GstD9, Gnmt, CG6300, CG11659, pug, Sardh, MsrA | 2.54 |
| GO:0006082 organic acid metabolic process | Nos, Sardh, Hpd, CG5955, CG3999, Gnmt, smp-30, CG6300, CG11659, yellow-h, Ddc, Gad1, pug, AANATL2, Kua, CG8665, CG6465, CG1544, hll, Nmdmc, MsrA, CG3699 | 2.25 |
| GO:0099536 synaptic signaling | Grd, nAChRbeta2, Syn, Snap25, Ir75a, cpx, Nlg4, Rbp, 5-HT1B, DopEcR, CG1909, Cadps, Shal, Tusp, CG31475, Rph, Syngr | 2.19 |
| GO:0019752 carboxylic acid metabolic process | Nos, Sardh. Hpd. CG5955, CG3999, Gnmt, smp-30, CG6300, CG11659, yellow-h, Ddc, Gad1, pug, AANATL2, Kua, CG8665, CG6465, CG1544, hll, Nmdmc, CG3699 | 2.19 |
| GO:0043436 oxoacid metabolic process | Nos, Sardh, Hpd, CG5955, CG3999, Gnmt, smp-30, CG6300, CG11659, yellow-h, Ddc, Gad1, pug, AANATL2, Kua, CG8665, CG6465, CG1544, hll, Nmdmc, CG3699 | 2.18 |
| GO:0099537 trans-synaptic signaling | Grd, nAChRbeta2, Syn, Snap25, Ir75a, cpx, Nlg4, Rbp, 5-HT1B, DopEcR, CG1909, Cadps, Shal, CG31475, Rph, Syngr | 2.12 |
| GO:0050808 synapse organization | dpr8, Nlg4, DIP-iota, Syn2, Rbp, Appl, Gad1, Syn, sff, plum, cpx, metro, CG31475, DIP-lambda, Nos | 2.07 |
| GO:0055085 transmembrane transport | Grd, nAChRbeta2, Mdr49, Shal, Mdr50, CG10804, CG8206, KCNQ, ppk12, CG1139, CG10226, Zip71B, Tim17a2, ppk3, ppk8, CG42340, CngB, Ca-alpha1T, Rgk1, bw, tipE, CG12531, CG3285, Sln, CG8468, TrpA1, Ir75a, ChT, CG17751, CG16727, CG3690, CG31272, CG33282, CG42269, Vha16-4, Tmc, shakB | 1.83 |
| GO:0003008 system process | Grd, nAChRbeta2, Or42b, robo3, Ca-alpha1T, Appl, Ddc, TrpA1, Ir75a, Mdr49, Mdr50, Cyp6w1, CG10226, CG6972, Rgk1, bw, Gad1, Syn, Shal, Nos, NPF, KCNQ, DopEcR, Desi, wry, Tmc, Obp84a, dpr8, CheB38b | 1.72 |

**Supplementary Table 2b: Down-regulated genes and their enriched biological processes with pathways and gene ontology ID**

| **Pathway** | **Genes** | **Fold Enrichment** |
| --- | --- | --- |
| GO:0042628 mating plug formation | Ebp, EbpII, Acp36DE | 46.98 |
| GO:0007618 mating | Acp26Aa, Cyp4d21 | 41.76 |
| GO:0045434 negative regulation of female receptivity post-mating | EbpII, Sems, intr, Dup99B, Acp36DE, Acp33A | 25.05 |
| GO:0007621 negative regulation of female receptivity | EbpII, Sems, intr, Dup99B, Acp36DE, Acp33A | 22.11 |
| GO:0019835 cytolysis | LysB, LysD LysE LysS | 17.89 |
| GO:0046008 regulation of female receptivity post-mating | Dup99B, EbpII, Sems, intr, Acp36DE, Acp33A | 17.08 |
| GO:0046693 sperm storage | Acp36DE, Sems, intr | 17.08 |
| GO:0044703 multi-organism reproductive process | Acp36DE, Acp26Aa, Ebp, Acp62F, Cyp4d21, Sems, intr, EbpII | 16.16 |
| GO:0007320 insemination | Acp36DE, Ebp, Acp62F, Sems, intr, EbpII, Acp26Aa | 14.62 |
| GO:0044706 multi-multicellular organism process | Acp36DE, Ebp, Acp62F, Sems, intr, EbpII, Acp26Aa | 14.62 |
| GO:0007620 copulation | Acp36DE, Ebp, Acp62F, Sems, intr, EbpII, Acp26Aa | 12.18 |
| GO:0045924 regulation of female receptivity | Dup99B, EbpII, Sems, intr, Acp36DE, Mst57Db, Acp33A | 11.86 |
| GO:0046692 sperm competition | Acp36DE, Acp62F, Sems, intr, Acp26Aa | 11.59 |
| GO:0018991 oviposition | BG642312, Dup99B, Mst57Db | 10.44 |
| GO:0060180 female mating behavior | Dup99B, EbpII, Sems, intr, Acp36DE, Mst57Db, Acp33A | 9.97 |
| GO:0018401 peptidyl-proline hydroxylation to 4-hydroxy-L-proline | CG18233, CG18231, PH4alphaPV, CG34041 | 9.28 |
| GO:0019511 peptidyl-proline hydroxylation | CG18233, CG18231, PH4alphaPV, CG34041 | 8.95 |
| GO:0018126 protein hydroxylation | CG18233, CG18231, PH4alphaPV, CG34041 | 7.83 |
| GO:0007617 mating behavior | Acp36DE, Acp26Ab, Dup99B, Ebp, Obp69a, EbpII, Acp62F, Obp56h, Sems, intr, Mst57Db, Acp33A, Acp26Aa | 5.62 |
| GO:0019098 reproductive behavior | Acp36DE, Acp26Ab, Dup99B, Ebp, Obp69a, EbpII, Acp62F, Obp56h, Sems, intr, BG642312, Mst57Db, Acp33A, Acp26Aa | 5.59 |
| GO:0010466 negative regulation of peptidase activity | Spn28F, Spn77Bb, Spn77Bc, Spn75F, Acp76A, Acp62F, CG3513, Sfp24Ba, Sfp24Bb | 5.47 |
| GO:0010951 negative regulation of endopeptidase activity | Spn28F, Spn77Bb, Spn77Bc, Spn75F, Acp76A, Acp62F, CG3513, Sfp24Ba, Sfp24Bb | 5.47 |
| GO:0045861 negative regulation of proteolysis | Spn28F, Spn77Bb, Spn77Bc, Spn75F, Acp76A, Acp62F, CG3513, Sfp24Ba, Sfp24Bb | 4.9 |
| GO:0051346 negative regulation of hydrolase activity | Spn28F, Spn77Bb, Spn77Bc, Spn75F, Acp76A, Acp62F, CG3513, Sfp24Ba, Sfp24Bb | 4.66 |
| GO:0052548 regulation of endopeptidase activity | Spn28F, Spn77Bb, Spn77Bc, Spn75F, Acp76A, Acp62F, CG3513, Sfp24Ba, Sfp24Bb | 4.18 |
| GO:0052547 regulation of peptidase activity | Spn28F, Spn77Bb, Spn77Bc, Spn75F, Acp76A, Acp62F, CG3513, Sfp24Ba, Sfp24Bb | 3.97 |
| GO:0043086 negative regulation of catalytic activity | Spn28F, Spn77Bb, Spn77Bc, Spn75F, Acp76A, Acp62F, CG3513, Sfp24Ba, Sfp24Bb | 3.32 |
| GO:0016042 lipid catabolic process | Yp1, CG4586, CG6296, CG34447, CG14034, CG18258, CG18301, CG17097 | 2.93 |
| GO:0044092 negative regulation of molecular function | Spn28F, Spn77Bb, Spn77Bc, Spn75F, Acp76A, Acp62F, CG3513, Sfp24Ba, Sfp24Bb | 2.76 |
| GO:0019953 sexual reproduction | Anp, Acp26Aa, Acp26Ab, Ebp, msopa, Acp36DE, Mst57Db, EbpII, Acp76A, Acp62F, Met75Cb, Spn28F, CG31883, Acp53C14a, Acp53C14b, CG15116, Obp56g, Spn77Bb, Spn77Bc, Sems, lectin-30A, BG642312, CG31872, Spn75F, CG34034, BG642163, Dup99B CG14034, Sfp24Ba, Sfp24Bb, Sfp60F, Sfp65A, Sfp77F, Sfp87B, Sfp26Ad, Sfp96F, CG43319, CG17097, Acp33A,CG18233, CG11131, intr, mei-218, gammaTub37C, Cyp4d21, hkb | 2.42 |
| GO:0032504 multicellular organism reproduction | Anp, Ebp, msopa, Mst57Db, EbpII, Acp62F, Met75Cb, CG31883, Acp53C14a, Acp53C14b, CG15116, Obp56g, Spn77Bb, Sems, lectin-30A, BG642312, Spn75F, CG34034, BG642163, Dup99B, Sfp24Ba, Sfp24Bb, Sfp60F, Sfp65A, Sfp77F, Sfp87B, Sfp26Ad, Sfp96F, CG43319, Acp33A, CG9988, Acp36DE, Acp26Ab, Acp26Aa, Spn28F, CG18233, Spn77Bc, CG11131, intr, CG31872, CG14034, CG17097, mei-218, gammaTub37C, Obp69a, Obp56h, hkb, CG11112 | 2.26 |
| GO:0000003 reproduction | Anp, Acp26Aa, Acp26Ab, Ebp, msopa, Acp36DE, Mst57Db, EbpII, Acp76A, Acp62F, Met75Cb, Spn28F, CG31883, Acp53C14a, Acp53C14b, CG15116, Obp56g, Spn77Bb, Spn77Bc, Sems, lectin-30A, BG642312, CG31872, Spn75F, CG34034, BG642163, Dup99B, CG14034, Sfp24Ba, Sfp24Bb, Sfp60F, Sfp65A, Sfp77F, Sfp87B, Sfp26Ad, Sfp96F, CG43319, CG17097, Acp33A, gammaTub37C, narya, CG9988, janB, CG18233, CG11131, intr, mei-218, Obp69a, CG11700, Cyp4d21, Obp56h, hkb,Yp1, CG11112 | 2.25 |
