# Supplementary material for: Diving Deeper Into Mechanisms of Acrylamide‐Induced Toxicity: RNA Sequencing Reveals Transcriptomic Alteration and Retrotransposon Expression in Drosophila melanogaster
Source: Environ Toxicol. 2026 Feb 23;41(10):1105–18. doi: 10.1002/tox.70054 (PMC13537343; doi:10.1002/tox.70054)
Supplement: Supplementary file 4 [file TOX-41-1105-s004.docx]

**Supplementary Table 1a: List of Up-regulated genes and their enriched molecular functions, including the pathway and their gene ontology ID.**

| **Pathway** | **Genes** | **Fold Enrichment** |
| --- | --- | --- |
| GO:0004477 methenyltetrahydrofolate cyclohydrolase activity | Nmdmc, pug | 42.71 |
| GO:0016594 glycine binding | CG3999, Gnmt | 42.71 |
| GO:0008559 ABC-type xenobiotic transporter activity | CG10226, Mdr49, Mdr50 | 32.03 |
| GO:0004486 methylenetetrahydrofolate dehydrogenase [NAD(P)+] activity | Nmdmc, pug | 28.48 |
| GO:0004488 methylenetetrahydrofolate dehydrogenase (NADP+) activity | Nmdmc, pug | 28.48 |
| GO:0004558 alpha-1,4-glucosidase activity | Mal-A2, Mal-A1, Mal-B1, Mal-A7, Mal-A8, Mal-A6 | 25.63 |
| GO:0032450 maltose alpha-glucosidase activity | Mal-A2, Mal-A1, Mal-B1, Mal-A7, Mal-A8, Mal-A6 | 25.63 |
| GO:0090599 alpha-glucosidase activity | Mal-A2, Mal-A1, Mal-B1, Mal-A7, Mal-A8, Mal-A6 | 21.36 |
| GO:0005248 voltage-gated sodium channel activity | Ca-alpha1T, tipE | 21.36 |
| GO:0019238 cyclohydrolase activity | Nmdmc, pug | 21.36 |
| GO:0042910 xenobiotic transmembrane transporter activity | CG10226, Mdr49, Mdr50 | 18.31 |
| GO:0015926 glucosidase activity | Mal-A2, Mal-A1, Mal-B1, Mal-A7, Mal-A8, Mal-A6 | 16.02 |
| GO:0015562 efflux transmembrane transporter activity | Mdr49, Mdr5, CG10226 | 16.02 |
| GO:0005246 calcium channel regulator activity | Rgk1, inaF-C, inaF-B | 14.24 |
| GO:0016500 protein-hormone receptor activity | Lgr1, Lgr4 | 12.21 |
| GO:0106389 ecdysteroid 22-kinase activity | CG1561, CG7135, CG16898, CG6908 CG11878, CG10513, CG10560, CG18765 CG31104, CG31288, CG32195 | 9.21 |
| GO:0016646 oxidoreductase activity acting on the CH-NH group of donors NAD or NADP as acceptor | Nmdmc, pug, CG8665 | 8.54 |
| GO:0016597 amino acid binding | CG3999, Gnmt | 8.54 |
| GO:0099106 ion channel regulator activity | tipE, Rgk1, inaF-C, inaF-B | 7.77 |
| GO:0016645 oxidoreductase activity acting on the CH-NH group of donors | Nmdmc, pug, CG8665, Sardh | 6.83 |
| GO:0016247 channel regulator activity | tipE, Rgk1, inaF-C, inaF-B | 6.57 |
| GO:0008227 G protein-coupled amine receptor activity | Octbeta3R, 5-HT1B, DopEcR | 6.1 |
| GO:0022843 voltage-gated cation channel activity | Shal, KCNQ, Ir75a, Ca-alpha1T | 5.69 |
| GO:0004467 long-chain fatty acid-CoA ligase activity | CG6300, CG11659, hll | 5.57 |
| GO:0004497 monooxygenase activity | Nos, Cyp4e2, Cyp9b1, Cyp6g1, Cyp28d2, Cyp6w1, Cyp4ad1, Cyp6g2, Cyp6a23, Cyp6a20, Cyp4aa1, Cyp6d5, Cyp12d1-p, Cyp12d1-d, Pal2 | 5.42 |
| GO:0008528 G protein-coupled peptide receptor activity | Lgr1, CG13579, AstA-R2, Lgr4, CCHa2-R, Octbeta3R | 5.34 |
| GO:0004714 transmembrane receptor protein tyrosine kinase activity | Ror, Ddr, otk | 5.34 |
| GO:0016814 hydrolase activity acting on carbon-nitrogen (but not peptide) bonds in cyclic amidines | Nmdmc, pug, CG12493 | 5.34 |
| GO:0015645 fatty acid ligase activity | CG6300, CG11659, hll | 4.93 |
| GO:0008188 neuropeptide receptor activity | CG13579, CCHa2-R, AstA-R2, Lgr1, Lgr4 | 4.75 |
| GO:0016405 CoA-ligase activity | CG6300, CG11659, hll | 4.75 |
| GO:0005272 sodium channel activity | ppk12, ppk3, ppk8, Ca-alpha1T, tipE | 4.64 |
| GO:0020037 heme binding | Gyc88E, Nos, Cyp4e2, Cyp9b1, Cyp6g1, Cyp28d2, Cyp6w1, Cyp4ad1, Cyp6g2, Cyp6a23, Cyp6a20, Cyp4aa1, Cyp6d5, Cyp12d1-p, Cyp12d1-d | 4.61 |
| GO:0046906 tetrapyrrole binding | Gyc88E, Nos, Cyp4e2, Cyp9b1, Cyp6g1, Cyp28d2, Cyp6w1, Cyp4ad1, Cyp6g2, Cyp6a23, Cyp6a20, Cyp4aa1, Cyp6d5, Cyp12d1-p, Cyp12d1-d | 4.58 |
| GO:0001653 peptide receptor activity | Lgr1, CG13579, AstA-R2, Lgr4, CCHa2-R, Octbeta3R | 4.49 |
| GO:0005244 voltage-gated ion channel activity | Shal, KCNQ, Ir75a, Ca-alpha1T | 4.17 |
| GO:0022832 voltage-gated channel activity | Shal, KCNQ, Ir75a, Ca-alpha1T | 4.17 |
| GO:0140359 ABC-type transporter activity | CG10226, bw, Mdr49, Mdr50 | 3.77 |
| GO:0004553 hydrolase activity hydrolyzing O-glycosyl compounds | Cht9, Mal-A2, Mal-A1, Mal-B1, Mal-A7, Mal-A8, Mal-A6, tobi | 3.72 |
| GO:0030594 neurotransmitter receptor activity | Grd, nAChRbeta2, Ir75a, 5-HT1B, DopEcR | 3.62 |
| GO:0016705 oxidoreductase activity acting on paired donors with incorporation or reduction of molecular oxygen | Nos, Cyp4e2, Cyp9b1, Cyp6g1, Cyp28d2, Cyp6w1, Cyp4ad1, Cyp6g2, Cyp6a23, Cyp6a20, Cyp4aa1, Cyp6d5, Cyp12d1-p, Cyp12d1-d, Pal2 | 3.56 |
| GO:0099094 ligand-gated cation channel activity | ppk12, Ir75a, ppk3, ppk8, CngB, nAChRbeta2 | 3.56 |
| GO:0005506 iron ion binding | Cyp4e2, Cyp9b1, Cyp6g1, Cyp28d2, Cyp6w1, Cyp4ad1, Cyp6g2, Cyp6a23, Cyp6a20, Cyp4aa1 Cyp6d5, Cyp12d1-p, Cyp12d1-d | 3.51 |
| GO:0004930 G protein-coupled receptor activity | Lgr1, CCHa2-R, CG13579, DopEcR, AstA-R2, Lgr4, Octbeta3R, 5-HT1B, CG43795 | 3.2 |
| GO:0005261 cation channel activity | Shal, KCNQ, ppk12, Ir75a, ppk3, ppk8, CG42340, Ca-alpha1T, CngB, Grd, TrpA1, nAChRbeta2, tipE | 3.17 |
| GO:0016798 hydrolase activity acting on glycosyl bonds | Cht9, Mal-A2, Mal-A1, Mal-B1, Mal-A7, Mal-A8, Mal-A6, tobi | 2.85 |
| GO:0015081 sodium ion transmembrane transporter activity | ppk12, ChT, ppk3, ppk8, Ca-alpha1T, CG10804, tipE | 2.85 |
| GO:0022836 gated channel activity | Shal, KCNQ, ppk12, Ir75a, ppk3, ppk8, Ca-alpha1T, CngB, Tmc, Grd, TrpA1, nAChRbeta2 | 2.66 |
| GO:0046873 metal ion transmembrane transporter activity | Shal, KCNQ, ppk12, Zip71B, ChT, ppk3, ppk8 CG42340 Ca-alpha1T TrpA1 CG10804 tipE | 2.48 |
| GO:0015276 ligand-gated ion channel activity | ppk12, Ir75a, ppk3, ppk8, CngB, Grd, TrpA1, nAChRbeta2 | 2.38 |
| GO:0022834 ligand-gated channel activity | ppk12, Ir75a, ppk3, ppk8, CngB, Grd, TrpA1, nAChRbeta2 | 2.38 |
| GO:0015267 channel activity | Grd, Shal, KCNQ, ppk12, Ir75a, ppk3, ppk8, shakB, CG42340, Ca-alpha1T, CngB, Tmc, TrpA1, nAChRbeta2, tipE | 2.29 |
| GO:0022803 passive transmembrane transporter activity | Grd, Shal, KCNQ, ppk12, Ir75a, ppk3, ppk8, shakB, CG42340, Ca-alpha1T, CngB, Tmc, TrpA1, nAChRbeta2, tipE | 2.29 |
| GO:0005216 ion channel activity | Grd, Shal, KCNQ, ppk12, Ir75a, ppk3, ppk8, shakB, CG42340, Ca-alpha1T, CngB, Tmc, TrpA1, nAChRbeta2, tipE | 2.28 |
| GO:0004252 serine-type endopeptidase activity | CG31267, l(2)k05911, CG8170, CG17475, CG5246, CG16710, CG11842, rho-5, CG30371, CG30375, CG31265, CG32260, Sp212, CG34290, CG30283 | 2.27 |
| GO:0008236 serine-type peptidase activity | CG31267, l(2)k05911, CG8170, CG17475, CG5246, CG3739, CG16710, CG11842, rho-5, CG30371, CG30375, CG31265, CG32260, Sp212, CG34290, CG30283 | 2.25 |
| GO:0022890 inorganic cation transmembrane transporter activity | Shal, KCNQ, ppk12, Zip71B, Ir75a, ChT, ppk3, ppk8, CG42340, Vha16-4, Ca-alpha1T, CngB, Grd, TrpA1, nAChRbeta2, CG10804, tipE | 2.16 |
| GO:0016491 oxidoreductase activity | MsrA, Nmdmc, Nos, pug, Sodh-1, Sgp, CG6012, CG8665, Sardh,CG12766, Hpd, CG5955, CG3999, GstD8, Cyp4e2, Cyp9b1, Cyp6g1, CG9360, Cyp28d2, Cyp6w1, Cyp4ad1, Cyp6g2, Cyp6a23, Cyp6a20, Cyp4aa1, CG6282, Cyp6d5, CG1544, CG3699, ND-B8, Cyp12d1-p, CG33177, Cyp12d1-d, Pal2 | 2.09 |
| GO:0038023 signaling receptor activity | Hr3, Grd, nAChRbeta2, Ror, Lgr1, CG1576, Or42b, CCHa2-R, CG13579, DopEcR, Ir75a, AstA-R2, Ddr, Nlg4, Lgr4, Octbeta3R, 5-HT1B, otk, CG43795, shakB | 2.05 |
| GO:0060089 molecular transducer activity | Hr3, Grd, nAChRbeta2, Ror, Lgr1, CG15765, Or42b, CCHa2-R, CG13579, DopEcR, Ir75a, AstA-R2, Ddr, Nlg4, Lgr4, Octbeta3R, 5-HT1B, otk, CG43795, shakB | 2.05 |
| GO:0008324 cation transmembrane transporter activity | Shal, KCNQ, ppk12, Zip71B, Ir75a, ChT, ppk3, ppk8, CG42340, Vha16-4, Ca-alpha1T, CngB, Grd TrpA1, nAChRbeta2, CG10804, tipE | 2.03 |
| GO:0017171 serine hydrolase activity | smp-30, CG17475, CG3739, CG31265, CG31267, l(2)k05911, CG8170, CG5246, CG16710, CG11842, rho-5, CG30371, CG30375, CG32260, Sp212, CG34290, CG30283 | 1.99 |
| GO:0004888 transmembrane signaling receptor activity | Ror, Lgr1, Or42b, CCHa2-R, CG13579, DopEcR, Ir75a, AstA-R2, Ddr, Lgr4, Octbeta3R, 5-HT1B, Grd, otk, nAChRbeta2, CG43795 | 1.97 |
| GO:0016773 phosphotransferase activity alcohol group as acceptor | Ror, CG4629, sff , Ddr, otk, IP3K2, CG1561, CG7135, CG16898, CG6908, CG11878, CG10513, CG10560, CG18765, CG31104, CG31288, CG32195 | 1.96 |
| GO:0022857 transmembrane transporter activity | Grd, Mdr49, Shal, Mdr50, CG12531, KCNQ, Sln, CG8468, ppk12, CG1139, CG10226, Zip71B, Ir75a, Tim17a2, ChT, ppk3, ppk8, shakB, CG42340, Vha16-4, Ca-alpha1T, CngB, Tmc, TrpA1, bw, nAChRbeta2, CG10804, CG3285, CG17751, CG16727, CG3690, CG31272, CG33282, CG42269, cpx, tipE | 1.87 |
| GO:0005215 transporter activity | Grd, Mdr49, Shal, Mdr50, CG12531, KCNQ, Sln, CG8468, ppk12, CG1139, CG10226, Zip71B, Ir75a, Tim17a2, ChT, ppk3, ppk8, shakB, CG42340, Vha16-4, Ca-alpha1T, CngB, Tmc, TrpA1, bw, nAChRbeta2, CG10804, CG3285, CG17751, CG16727, CG3690, CG31272, CG33282, CG42269, cpx, tipE | 1.76 |
| GO:0015318 inorganic molecular entity transmembrane transporter activity | Grd, Shal, KCNQ, ppk12, Zip71B, Ir75a, ChT, ppk3, ppk8, CG42340, Vha16-4, Ca-alpha1T, CngB, Tmc, TrpA1, nAChRbeta2, CG10804, tipE | 1.68 |
| GO:0008233 peptidase activity | CG17636, CG3739, CG31267, nolo, CG32379, l(2)k05911, CG8170, CG6337, CG10062, CG5282, Nepl12, CG17475, CG5246, CG16710, CG11842, rho-5, CG30043, CG30371, CG30375, CG31265, CG32260, Sp212, CG34290, CG30283 | 1.68 |
| GO:0003824 catalytic activity | smp-30, CG17475, CG3739, CG31265, Ddc, MsrA, Gad1, GstD8, Nmdmc, Ror, Nos, pug, Sodh-1, CG8910, CG17636, CG14443, hdm, CG10352, CG4629, AANATL2, Sgp, CG6012, CG8665, Dgat2, CG1946, CG12913, Sardh, Cht9, Alp8, CG16758, CG12766, CG12493, sff, Hpd, CG5955, CG11425, CG3999, CG6465, GstD9, Gnmt, Gyc88E, CG6300, CG11659, CG31267, nolo, CG32379, Ddr, IP3K2, l(2)k05911, otk, Scp2, Lip3, yellow-h, Pal2, hll, bw, Mal-A2, Mal-A1, Mdr49, Mdr50, Cyp4e2, Cyp9b1, Cyp6g1, Ugt37B1, Ugt36F1, CG9360, CG2772, Cyp28d2, CG11149, CG14535, Mal-B1, CG10383, Cyp6w1, Cyp4ad1, Mal-A7, Mal-A8, CG8170, Cyp6g2, CG6337, Cyp6a23, Cyp6a20, CG8093, Cyp4aa1, CG10062, CG10226, CG8641, CG6282, CG5282, CG16789, Nepl12, Cyp6d5, CG5246, CG10182, CG16710, CG11842, CG1544, CG3699, ND-B8, rho-5, CG30043, Mal-A6, CG30371, CG30375, Cyp12d1-p, CG31091, CG32260, CG33177, Sp212, Cyp12d1-d, CG34290, CG30283, tobi, Rgk1, CG1561, CG7135, CG16898, CG6908, CG11878, CG10513, CG10560, CG18765, CG31104,G31288, CG32195 | 1.24 |

**Supplementary Table 1a: List of Down-regulated genes and their enriched molecular functions, including the pathway and their gene ontology ID.**

| **Pathway** | **Genes** | **Fold Enrichment** |
| --- | --- | --- |
| GO:0003796 lysozyme activity | LysD, LysB, LysE, LysS | 14.74 |
| GO:0004656 procollagen-proline 4-dioxygenase activity | CG18233, CG18231, PH4alphaPV, CG34041 | 9.64 |
| GO:0019798 procollagen-proline dioxygenase activity | CG18233, CG18231, PH4alphaPV, CG34041 | 9.64 |
| GO:0031545 peptidyl-proline 4-dioxygenase activity | CG18233, CG18231, PH4alphaPV, CG34041 | 9.28 |
| GO:0031543 peptidyl-proline dioxygenase activity | CG18233, CG18231, PH4alphaPV, CG34041 | 8.95 |
| GO:0004806 triglyceride lipase activity | Yp1, CG6296, CG34447, CG14034, CG18258, CG18301, CG31872, CG17097 | 8.21 |
| GO:0061783 peptidoglycan muralytic activity | LysD, LysB, LysE, LysS | 8.08 |
| GO:0016702 oxidoreductase activity acting on single donors with incorporation of molecular oxygen incorporation of two atoms of oxygen | CG18233, CG18231, PH4alphaPV, CG34041 | 7.37 |
| GO:0016701 oxidoreductase activity acting on single donors with incorporation of molecular oxygen | CG18233, CG18231, PH4alphaPV, CG34041 | 7.16 |
| GO:0008061 chitin binding | CG7298, CG7290, CG7017, CG6933, CG7714, CG7715, CG13643, CG32302, CG17147, CG42729, CG43673, CG43896, Vajk3, CG30281 | 7.02 |
| GO:0004867 serine-type endopeptidase inhibitor activity | Spn77Bb, Spn77Bc, Spn75F, Acp62F, Acp76A, Spn28F, CG3513, Sfp24Ba, Sfp24Bb | 6.79 |
| GO:0031418 L-ascorbic acid binding | CG18233, CG18231, PH4alphaPV | 6.06 |
| GO:0080030 methyl indole-3-acetate esterase activity | Yp1, CG34447, CG14034 | 5.87 |
| GO:0004866 endopeptidase inhibitor activity | Spn77Bb, Spn77Bc, Spn75F, Acp62F, Acp76A, Spn28F CG3513, Sfp24Ba, Sfp24Bb | 5.64 |
| GO:0061135 endopeptidase regulator activity | Spn77Bb, Spn77Bc, Spn75F, Acp62F, Acp76A, Spn28F CG3513, Sfp24Ba, Sfp24Bb | 5.53 |
| GO:0030414 peptidase inhibitor activity | Spn77Bb, Spn77Bc, Spn75F, Acp62F, Acp76A, Spn28F CG3513, Sfp24Ba, Sfp24Bb | 5.47 |
| GO:0031406 carboxylic acid binding | CG4586, CG18233, CG18231, PH4alphaPV | 5.45 |
| GO:0016298 lipase activity | Yp1, CG6296, CG34447, CG14034, CG18258, CG18301, CG31872, CG17097 | 5.22 |
| GO:0061134 peptidase regulator activity | Spn77Bb, Spn77Bc, Spn75F, Acp62F, Acp76A, Spn28F CG3513, Sfp24Ba, Sfp24Bb | 5.13 |
| GO:0016706 2-oxoglutarate-dependent dioxygenase activity | CG18233, CG18231, PH4alphaPV, CG34041 | 4.64 |
| GO:0048029 monosaccharide binding | CG18233, CG18231, PH4alphaPV, lectin-30A | 4.25 |
| GO:0004857 enzyme inhibitor activity | Spn77Bb, Spn77Bc, Spn75F, Acp62F, Acp76A, Spn28F, CG3513, Sfp24Ba, Sfp24Bb | 4.12 |
| GO:0051213 dioxygenase activity | CG18233, CG18231, PH4alphaPV, CG34041 | 3.98 |
| GO:0052689 carboxylic ester hydrolase activity | Yp1, CG6296, CG34447, CG14034, CG18258, CG18301, CG31872, CG17097 | 3.34 |
| GO:0005214 structural constituent of chitin-based cuticle | Cpr97Ea, Ccp84Ab, Cpr31A, CG34461, Vajk3,TwdlE, Twdlalpha | 2.96 |
| GO:0017171 serine hydrolase activity | Yp1,epsilonTry,Jon25Bii,CG18301,CG18179, CG18180,Sems,CG31872,CG33096,CG34447, CG17097,CG18258,Ser8,CG9377,intr,2CG30098, CG33459 | 2.92 |
| GO:0016705 oxidoreductase activity acting on paired donors with incorporation or reduction of molecular oxygen | CG18233,CG18231,CG9747, PH4alphaPV, CG34041 Cyp4d21, Cyp312a1, Cyp304a1 | 2.78 |
| GO:0005506 iron ion binding | CG9747, Cyp4d21, Cyp312a1, CG18233, CG18231, Cyp304a1, PH4alphaPV | 2.78 |
| GO:0042302 structural constituent of cuticle | Cpr97Ea, Ccp84Ab, Cpr31A, CG34461, Vajk3, TwdlE, Twdlalpha | 2.74 |
| GO:0004252 serine-type endopeptidase activity | epsilonTry, Ser8, Sems, Jon25Bii, CG9377, CG18179, CG18180, intr, CG30098, CG33459 | 2.22 |
